# Analysis of multiplexed SERS-based lateral flow assays: turning qualitative into quantitative for inflammatory bowel disease monitoring

**DOI:** 10.1039/d6an00820h

**Published:** 2026-08-18

**Authors:** Jodie Fergusson, Gregory Q. Wallace, Sian Sloan-Dennison, Neil C. Shand, Duncan Graham, Karen Faulds

**Affiliations:** a Centre for Nanometrology, Department of Pure and Applied Chemistry, Technology and Innovation Centre 99 George Street Glasgow G1 1RD UK karen.faulds@strath.ac.uk; b Defence Science and Technology Laboratory Porton Down Salisbury SP4 0JQ UK

## Abstract

The development of diagnostic tests that offer rapid and quantitative results can improve the health of patients, suffering from both chronic and acute diseases. Inflammatory bowel disease (IBD) is a chronic autoimmune condition, where a patient can undergo periods of sudden flare-ups and prolonged remission. The current biomarkers of interest are either detected using central laboratory methods, or, are non-specific to IBD. In this work, we develop a series of lateral flow immunoassays for the detection of IBD biomarkers lactoferrin and myeloperoxidase, in an artificial stool matrix. By utilising surface enhanced Raman scattering (SERS) active nanotags, we demonstrate the feasibility of these SERS-based lateral flow immunoassays for proof-of-concept singleplex and duplex detection *via* Raman mapping. Due to the variation in background caused by the stool matrix, we have investigated methods of assessing the SERS signal from the Raman maps, to transition the tests from giving qualitative to quantitative results. This has been achieved by extracting the SERS intensities from the maps associated with non-specific interactions, to define unique intensity thresholds for each lateral flow immunoassay, overall improving the sensitivity, reproducibility, and linearity in limit of detection studies. We achieved limits of detection of 3.2 ng mL^−1^ for lactoferrin and 5.0 ng mL^−1^ for myeloperoxidase, and limits of quantification of 18.8 ng mL^−1^ for lactoferrin and 8.0 ng mL^−1^ for myeloperoxidase. These values are well below the “normal” level of these biomarkers in stool, indicating this assay would be able to accurately monitor changing biomarker concentrations. Furthermore, we used a ratiometric approach to evaluate the maps from duplex lateral flows and detect each biomarker simultaneously on the same strip. Overall, this work demonstrates that it is possible to quantitatively detect IBD biomarkers in stool on a lateral flow with SERS, using improved Raman signal analysis methods to overcome complex sample background interference and enhance sensitivity.

## Abstract 2

Investigation into different methods to assess the SERS signal from lateral flow immunoassay tests for single and duplex detection of inflammatory bowel disease biomarkers.

## Graphical abstract

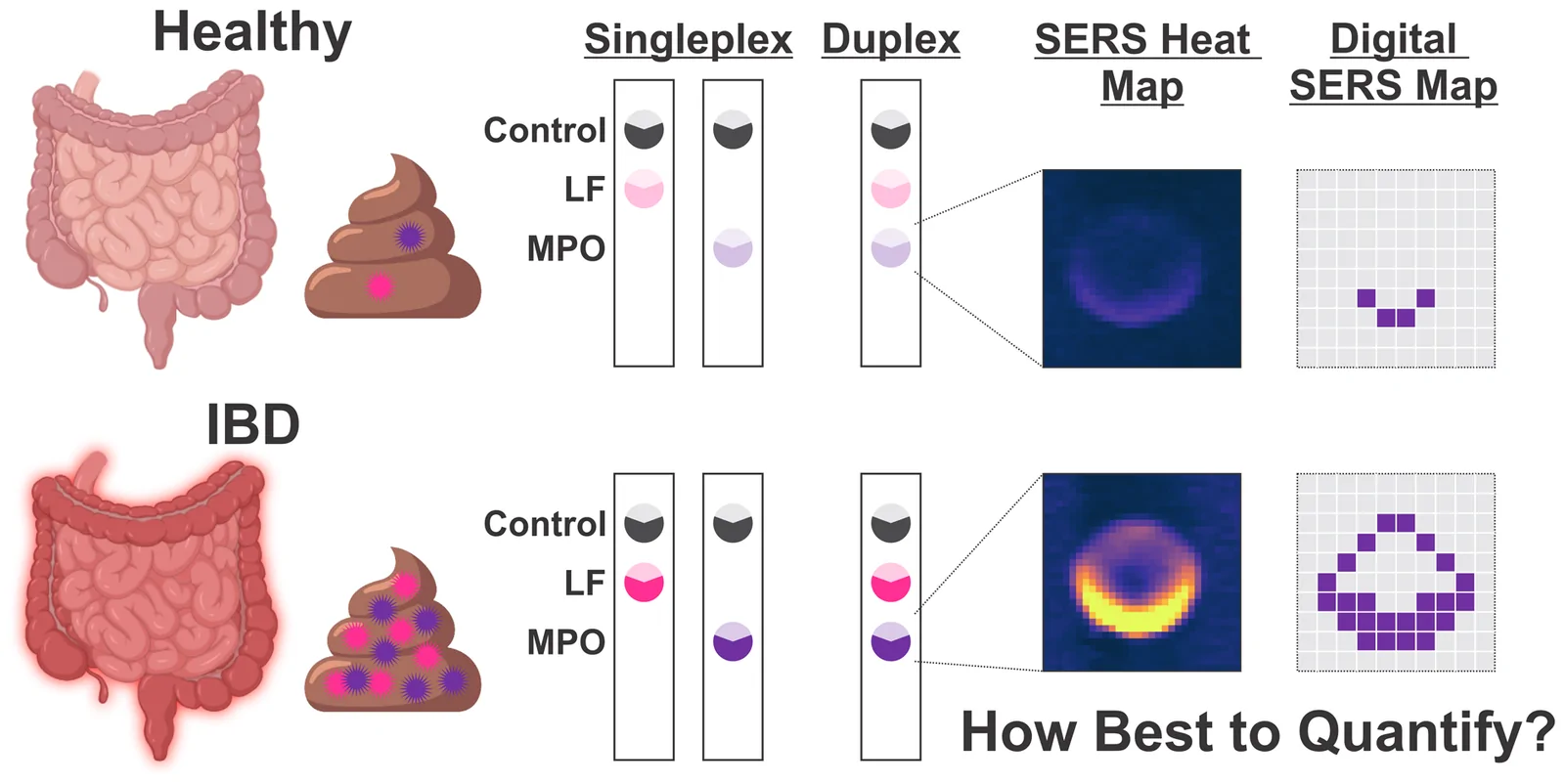

## 1. Introduction

Inflammatory bowel disease (IBD) is a chronic autoimmune condition, currently affecting 1 in 123 people in the United Kingdom.^1^ In this condition, the immune system attacks healthy bacteria in the intestine, causing inflammation.^2^ IBD can present itself in several ways, with the two most common forms being ulcerative colitis and Crohn's disease.^3^ Ulcerative colitis is characterised by continuous inflammation spreading from the rectum along the large intestine only, whilst Crohn's disease results in patchy inflammation occurring anywhere between the oesophagus and rectum.^4^ IBD occurs in periods of flare-ups and remission, where flare-ups can lead to complications ranging from rectal bleeding and abdominal pain,^5^ to fistulas^6^ and toxic megacolon,^7^ the latter being a life-threatening complication requiring immediate surgery.

Disease activity is typically monitored using a combination of colonoscopy,^8^ and enzyme-linked immunosorbent assays (ELISAs) to determine the concentration of the biomarker faecal calprotectin present in stool.^9^ A serum biomarker that can be monitored to evaluate IBD activity is C-reactive protein (CRP);^10^ however, elevated CRP can be indicative of several conditions, such as infection or sepsis,^11^ and serum biomarkers are less likely to reflect the degree of inflammation within the intestine, in comparison to stool biomarkers.^10^ Two alternative biomarkers which present at similar clinical concentration ranges in stool are faecal lactoferrin (LF)^12^ and myeloperoxidase (MPO),^13^ which could offer critical insights into disease activity. LF is especially useful as it strongly correlates with intestinal inflammation,^14,15^ thus helping to distinguish IBD from functional disorders, such as irritable bowel syndrome.^16^ However, the challenge not only lies with identifying the correct biomarkers(s), but with the tests used. Although ELISAs are reliable and quantitative, there is often a delay between sample collection, testing in a laboratory, and results being given, during which time disease activity can fluctuate, or a patient's condition may worsen and require hospitalisation.^17^ There is a need to monitor disease activity in real-time, allowing patients to predict impending flare-ups sooner, and allowing healthcare providers to administer treatment before any irreversible effects of flare-ups occur.

Lateral flow immunoassays (LFIAs) are a low-cost and simple alternative to lab-based ELISAs.^18^ They used labelled antibodies to specifically capture a target biomarker in a sample. Once bound, the complex flows along a strip to a test zone, where a detection antibody binds to the complex, forming a sandwich assay and immobilising the label. The label can be latex beads, or a fluorophore, but most commonly, gold nanoparticles are used, which allows visual identification of a red coloured line at the test zone.^19^ Typically, LFIAs have been associated with over-the-counter pregnancy tests,^20^ but in recent years, the public have become familiar with them *via* the rapid antigen tests for SARS-CoV-2, with over 3 billion tests being used between 2020 and 2022.^21^ LFIAs are particularly attractive as point-of-care (POC) tests as they comply with the World Health Organisation's “ASSURED” criteria, which states that POC tests should be affordable, sensitive, specific, user-friendly, rapid/robust, equipment-free, and deliverable.^22^ However, despite their advantages, LFIAs possess some drawbacks; mainly, that their test results are qualitative, or semi-quantitative at best, achieved by visually interpreting the intensity of the coloured test line.^23^ Interpretation can be negatively impacted by the sample matrix, with bodily fluids such as blood and stool making it difficult to determine the colour of the test line.^24^ For LFIAs to be fully quantitative, it is necessary to couple them with a read-out technique which is label dependent. This includes, but is not limited to, fluorescence,^25^ electrochemical sensing,^26^ and of greater interest, surface enhanced Raman scattering (SERS).^27^

To couple LFIAs with quantitative SERS measurements, the label must be a nanoparticle functionalised with a Raman reporter molecule. Many different nanoparticle platforms have been developed with a range of protective coatings to minimise aggregation when exposed to complex matrices, such as serum or stool, during LFIA testing. One of the most successful designs consists of gold nanoparticles functionalised with a Raman reporter and encapsulated within a silica shell, generally referred to as a shell-isolated nanoparticle (SHIN). The silica shell has been shown to protect the nanoparticle from aggregation in serum, enabling them to retain their size, shape, and SERS signal, following serum exposure.^28^

The test line can then be interrogated with a laser, and the SERS spectrum of the Raman reporter detected.^29^ The intensity of the SERS signal can be correlated to the concentration of the biomarker present. SERS-based LFIAs have been used to detect and quantify HIV-1,^30^ staphylococcal enterotoxin B,^31^ cardiac troponin,^32^ and more recently, cytokeratin-18,^33^ a biomarker for drug induced liver injury. In these examples, only one biomarker was detected; however, multiplex detection can also be achieved with conventional LFIAs, as well as SERS-based LFIAs.^34^

Multiplexed SERS-LFIAs can be spatially or spectrally resolved.^29^ In a spatially resolved configuration, multiple test lines are arranged along a strip, with each line designed to capture a different biomarker.^34^ By analysing each test line, the levels of corresponding biomarkers can be quantified. Often, the same Raman reporter label is used for each biomarker, so each line produces the same SERS signal. Whilst this approach is effective, it does not provide information on potential non-specific binding or cross-reactivity between biomarkers. Multiplexed LFIAs can also be spectrally resolved. In this approach, the test line is composed of multiple antibodies, allowing several biomarkers to be detected on the same line.^35^ To distinguish which biomarker is present, the labels need to be different. SERS is particularly well-suited for this application, as the Raman reporter labels can be chosen to be spectrally distinct and easily identifiable from one another. Multiplexed SERS-LFIAs have shown promise for the rapid diagnosis of clostridium difficile in synthetic stool samples based on the presence of different surface layer proteins,^36^ as well as the presence of histamine and parvalbumin in fish.^37^

Transitioning to quantitative LFIA multiplex assays requires developing improved detection strategies for quantifying the biomarkers of interest. To this end, we have explored both singleplex and duplex detection of the IBD biomarkers LF and MPO using SERS-LFIAs. We have chosen not to include the “gold standard” biomarker calprotectin at this stage, as it presents at a higher concentration in stool compared to LF and MPO.^38^ Incorporating it would have required modifying the lateral flow assay to operate across two distinct concentration ranges, which would introduce complexity and potential variation in assay performance. These proof-of-concept assays employ a “dipstick format” to emphasise the importance of understanding and minimising non-specific binding, as well as strategies for its elimination. In this configuration, the sample pad and conjugate pad are absent, and the nitrocellulose strip is dipped into a vial containing a mixture of synthetic stool sample, buffer, nanotags, and the biomarker of interest. Importantly, we explore and discuss various means of processing the acquired SERS maps to generate calibration curves for singleplex detection, as well as demonstrate a ratiometric approach towards achieving multiplex detection of LF and MPO in an artificial stool matrix. Ultimately, this work offers an exciting new potential for the rapid monitoring of IBD flare-ups and forms the foundation for the development of a future sensitive and specific diagnostic tool.

## 2. Materials and methods

Lactoferrin and myeloperoxidase antibody pairs were purchased from Abcam, lactoferrin protein was purchased from BioRad, and myeloperoxidase protein was purchased from R&D Systems. Synthetic stool was purchased from Claremont Bio. Components for the lateral flow strips were purchased from Cytiva. Phosphate buffer saline (PBS) tablets were purchased from Oxoid. All remaining chemicals were purchased from Sigma Aldrich (Merck).

### 2.1. Buffer and sample matrix preparation

Borate buffer was prepared by dissolving 76 mg of sodium tetraborate and 15 mg boric acid in 10 mL of doubly distilled deionised water (ddd.H_2_O), and the mixture was heated at 50 °C until fully dissolved. Lateral flow buffer was prepared by dissolving one PBS tablet in 100 mL of ddd.H_2_O to give a PBS stock solution. To 10 mL of this, 100 mg of bovine serum albumin (BSA), and 10 µL of Tween20 was then added. Synthetic stool was purchased as a solid powder containing human serum albumin, and as per product instructions, dissolved in 5 mL of PBS buffer.

### 2.2. Gold nanoparticle synthesis

Gold nanoparticles were prepared *via* the Turkevich method.^39^ Prior to synthesis, a three-necked round bottom flask and glass stirrer rod were soaked in aqua regia, then rinsed with ddd.H_2_O. 60.5 mg of sodium tetrachloroaurate (NaAuCl_4_) was added to 500 mL of ddd.H_2_O in the flask and heated to boiling using a Bunsen burner. Once boiling, sodium citrate (Na_3_C_6_H_5_O_7_, 57.5 mg in 7.5 mL ddd.H_2_O) was added and the mixture heated for a further 15 minutes, before being left to cool with stirring overnight.

### 2.3. Shell-isolated nanoparticle preparation

Shell isolated nanotags (SHINs) were prepared using a method adapted from the protocol originally reported by Li *et al.*^40,41^ 50 mL of the synthesised gold nanoparticles were added to a round bottom flask with stirring, followed by either 1,2-bis(4-pyridyl)ethylene (BPE, 150 µL, 100 µM), or 4(1-*H*-pyrazol-4-yl)pyrimidine (PPY, 150 µL, 100 µM), to give a final Raman reporter concentration of 300 nM. 75 µL of (3-aminopropyl)trimethoxysilane (APTMS) (0.02%) and 750 µL of sodium silicate (20%) were then added in rapid succession to render the gold surface vitreophilic and allow the growth of a thin silica shell. The mixture was heated at 90 °C for 30 minutes and cooled overnight, both with constant stirring.

### 2.4. BPE-LF and PPY-MPO nanotag preparation

100 µL of borate buffer was added to 1 mL of the prepared SHINs, along with 2 µL of detection antibody (1 mg mL^−1^). BPE SHINs were functionalised with LF antibodies to produce BPE-LF nanotags, and PPY SHINs were functionalised with MPO antibodies to produce PPY-MPO nanotags. These were left to shake for two hours. After two hours, BSA (100 µL, 10 mg mL^−1^) was added to each, and the mixtures shaken for a further 30 minutes. To confirm the antibody had successfully functionalised the surface of the nanotags, they were characterised using extinction spectroscopy and dynamic light scattering (DLS). The Ab-functionalised nanotags were then centrifuged at 4000 rpm for 20 minutes, the supernatant removed, and the pellet resuspended in 100 µL of ddd.H_2_O.

### 2.5. Strip preparation

Lateral flow strips were prepared by cutting nitrocellulose (NC) strips to 0.5 cm in width and assembling with an absorbent pad at the top. For singleplex concentration studies, the strips were spotted with a control spot of 0.5 µL of goat anti-rabbit antibody, diluted to 1 mg mL^−1^, using PBS buffer. Below this, 0.5 µL of either 1 mg mL^−1^ LF capture antibody, or 1 mg mL^−1^ MPO capture antibody, was spotted onto the NC strip as the test spot. For multiplex detection, strips were prepared by again spotting a control spot of 0.5 µL of 1 mg mL^−1^ goat anti-rabbit, and below this, 0.5 µL of LF capture antibody and 0.5 µL of MPO capture antibody for the test spots, with ∼0.5 cm spacing between each of the spots.

### 2.6 Sample mixture preparation and analysis

Sample mixtures were prepared in 1.75 mL glass vials, using the volumes shown in Table S1 for singleplex experiments, and the volumes shown in Table S2 for multiplex experiments. Once the sample mixtures had been prepared, the lateral flow strips were dipped into the glass vials as shown in Fig. S1 and left in place until the entirety of the sample volume had flowed up the length of the strip. The strips were then allowed to fully dry for 20 minutes, before analysis using Raman mapping.

### 2.7 Raman mapping and processing

Raman maps were acquired using a Renishaw InVia microscope. Strips were placed inside the sample chamber and the area for analysis selected using the camera. A 5× objective lens was used, a 100 µM step size was chosen, along with 785 nm excitation, 0.5 s acquisition time, and 5% laser power. The laser spot size was 8 µm. The resulting spectra were processed using WiRE. Within the WiRE software, a polynomial baseline correction was applied to all spectra within the maps. The processed spectra were then further analysed using Python scripts. SERS intensity maps were generated by finding the maximum peak intensities for BPE at ∼1640 cm^−1^, and for PPY at ∼955 cm^−1^. A tolerance in the peak position ensured that the maximum intensity value was used. Thresholding was performed by defining either fixed values, or calculated values based on the selection of background pixels in the map, corresponding to the four corners of the map and an area just before the spot. In total, 125 pixels were used. For any given pixel, if its value was above the threshold, it was kept. Otherwise, the value was set to 0 and subsequently ignored for quantitation. For duplex measurements, ratiometric maps were generated using the intensity at 955 cm^−1^ divided by the intensity at 1640 cm^−1^.

## 3. Results and discussion

### 3.1 Design of singleplex SERS-LFIAs

**Fig. 1:**
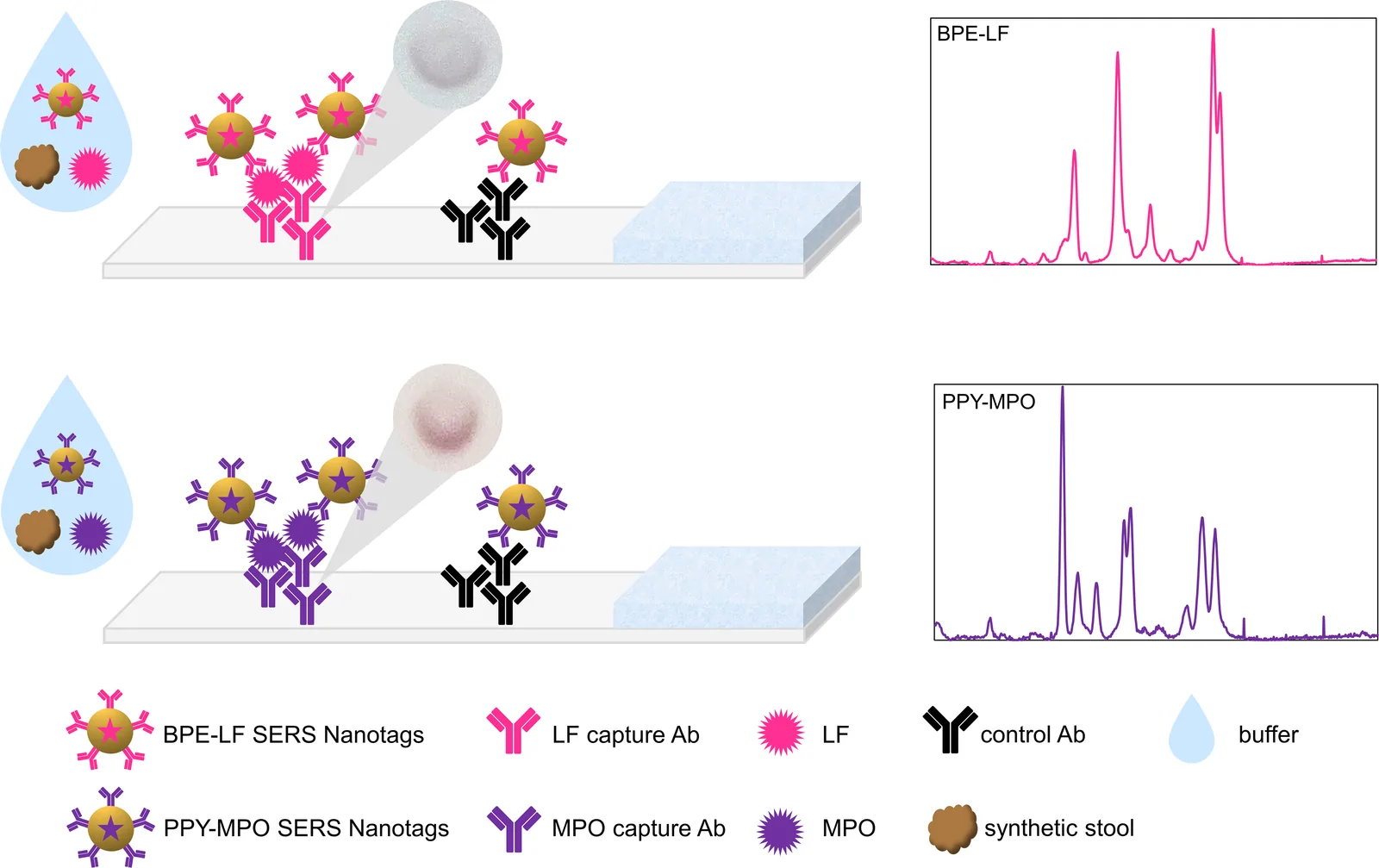
Schematic of SERS-LFIA tests for the singleplex detection of lactoferrin (LF) and myeloperoxidase (MPO) in synthetic stool matrix. Insert shows the representative SERS spectra of BPE-LF and PPY-MPO nanotags, collected using 785 nm laser excitation.

The concept and design of the two singleplex SERS-LFIAs are shown in Fig. 1. In singleplex form, the lateral flow has either an LF or MPO antibody at the test spot, and a goat anti-rabbit control antibody spot. Although test and control lines are more commonly used in commercial applications, the use of spots is a more cost-effective way of ascertaining the potential effectiveness of an assay during the development stages of a LFIA. This is due to less antibody being required to create a spot compared to a line, as well as not requiring any additional, relatively expensive instrumental to produce the spot. Test and control spots were prepared by dropping 0.5 µL of antibody onto the nitrocellulose, using a pipette. To build SERS-LFIAs for the detection of LF and MPO, two “flavours” of SERS nanotag were prepared; one to target LF, and another to target MPO. Each nanotag was composed of a gold nanoparticle core, functionalised with a unique Raman reporter, and then encapsulated in a silica shell. This is referred to as a shell-isolated nanoparticle (SHIN). A biomarker-specific antibody was then conjugated to the silica shell, creating a SERS nanotag. Combining each biomarker with a distinct Raman reporter creates spectrally unique nanotags, enabling the identification of the specific biomarker within the test zone based on the Raman reporter's unique spectral signature. 1,2-Bis(4-pyridyl)ethylene (BPE) was used for the LF specific SERS nanotags, and 4(1-*H*-pyrazol-4-yl)pyrimidine (PPY) for the MPO SERS nanotags. The SERS spectrum for each nanotag is shown in Fig. 1. Complete characterisation of the SERS nanotags is included in the SI and Fig. S2. The results indicate that the SERS nanotags produced strong and distinct BPE and PPY spectra, and that the conjugation of the biomarker antibody did induce unwanted aggregation.

To run the assays, the SERS nanotags were combined with the sample, which consisted of the synthetic stool matrix, spiked with different concentrations of biomarker, and running buffer, in a dipstick configuration as shown in Fig. S1. In this method, there are no sample and conjugate pads present on the strip, and the nanotags are added to the sample mixture rather than being immobilised on the strip. The lateral flow strip was then added to the mixture and allowed to run for 20 minutes. In the presence of the biomarker, a sandwich immunoassay formed on the test spot, immobilising the SERS nanotag, and indicating a positive response. A control spot, which consisted of goat anti-rabbit antibodies which bind to the antibody on the SERS nanotag, was used to ensure the LFIA was running correctly. Interrogating the test and/or control spots with a Raman microscope allows for the SERS spectrum of the SERS nanotag to be observed.

### 3.2. Generation of singleplex calibration curves

To assess the performance of the singleplex SERS-LFIAs, a triplicate limit of detection study was carried out by spiking synthetic stool with a series of biomarker concentrations (0, 5, 10, 15, 20, 25, 50, 75, and 100 ng mL^−1^). Once the sample solution had flowed through the strip, it was left to dry, and the visual results of the concentration studies are shown in Fig. S3. As expected, the visual intensity of the test spot increased with the concentration of each biomarker. Each test spot was then analysed *via* Raman mapping using 785 nm laser excitation and the results evaluated by creating heat maps and a calibration curve, relating the concentration of the biomarker to a heat map output. Representative SERS intensity heat maps are shown in Fig. S4. Again, as expected, the SERS intensity increased with the concentration of each biomarker. Both visual (Fig. S3) and SERS mapping (Fig. S4) highlighted challenges that needed to be considered: (i) the variation in size and shape of the test spots, (ii) the appearance of a crescent moon-like distribution of the SERS nanotags within the test spots, and (iii) varying background SERS signals outside of the test spots. The first two issues relate to how the antibody dries, and therefore how the sandwich assay forms during the assay, whilst the third relates to non-specific interactions. It was therefore necessary to develop a robust approach to generate quantitative calibration curves when using irregular and anisotropic spots.

**Fig. 2:**
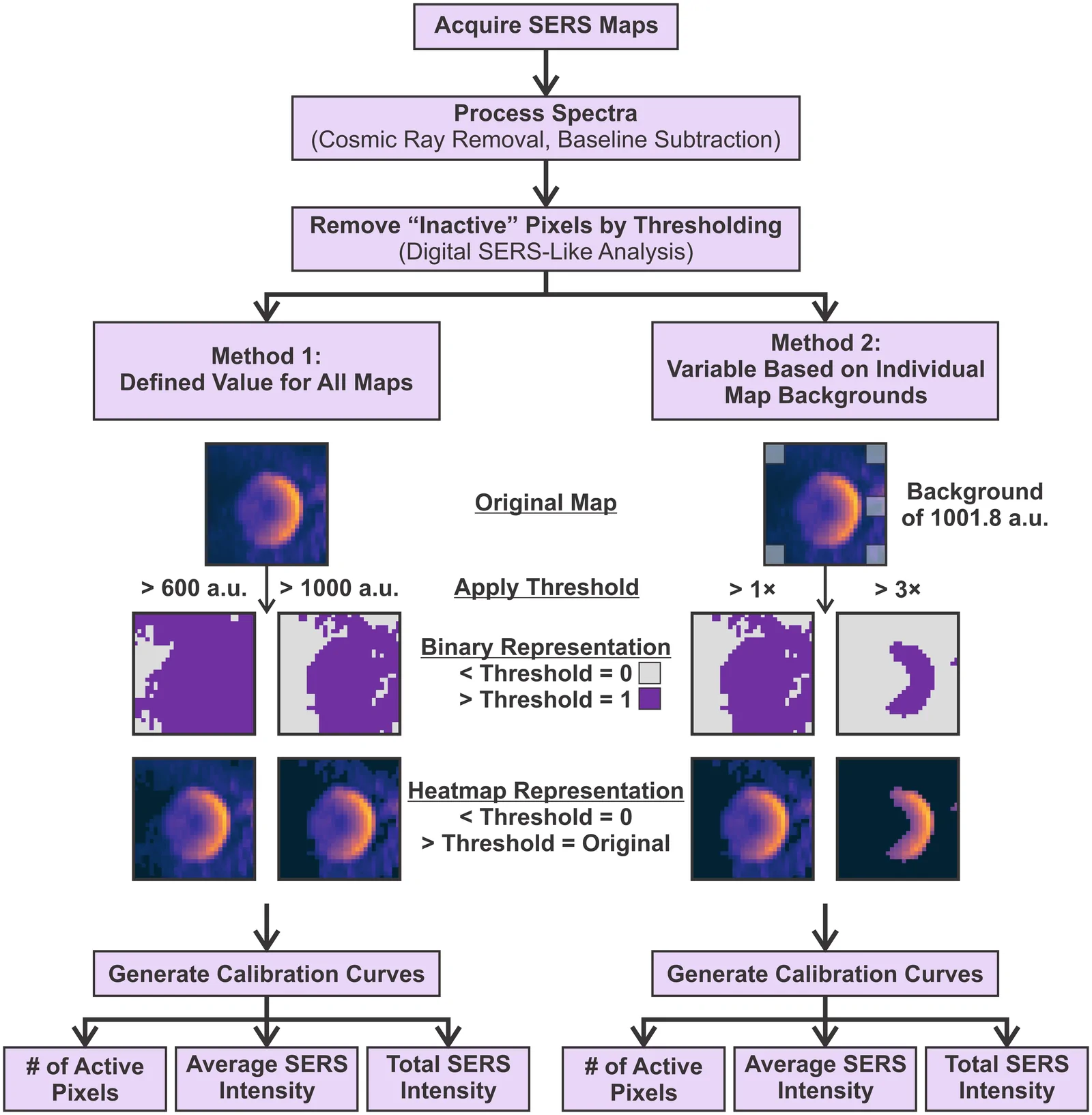
Workflow diagram for the processing of SERS maps to generate calibration curves for SERS-LFIAs. After acquiring the SERS maps, the spectra were processed as needed (cosmic ray removal, baseline subtraction). Removing the inactive pixels was achieved by either Method 1, using a defined value, or Method 2, using a variable value dependent on the background SERS signal of that map. Representative examples are shown for a SERS map for one of the three replicates of 50 ng mL^−1^ MPO, highlighting the distribution of active pixels and the revised SERS heatmaps. Calibration curves were subsequently generated by utilising different strategies: (i) the number of active pixels, (ii) the average SERS intensity of the active pixels, and (iii) the total SERS intensity of the active pixels.

As shown in Fig. 2, two methods for determining “active” pixels were considered: applying a fixed value background threshold to all SERS maps for the same biomarker (Method 1) and applying a variable background threshold based on the unique backgrounds from each SERS map (Method 2). Anything below this threshold was discarded. The remaining spectra were then used to generate binary maps to visualise the distribution of active pixels located within each map, as well as new SERS intensity maps that only considered the active pixels. SI Note S1, along with Fig. S5 and S6, provide more information on how the two methods for determining active pixels compare with one another. Ultimately, it was found that accounting for the unique backgrounds of each SERS map best minimised the incorporation of pixels related to non-specific interactions when generating the calibration curves.

The next challenge was to establish the calibration metric. Three options were considered: (i) the total number of active pixels within the map, (ii) the average SERS intensity of the active pixels, and (iii) the total SERS intensity of the active pixels. With two methods for identifying active and inactive pixels, five thresholds per method, three calibration metrics, and two biomarkers, a total of 60 calibration curves were generated (Fig. S7–10). A summary of the relative standard deviations, *R*^2^ values, and linear ranges is included in Tables S3 and S4. SI Note S2 and Fig. S11 offer greater insights into the decision to proceed with using the total SERS intensity of active pixels as the preferred strategy.

**Fig. 3:**
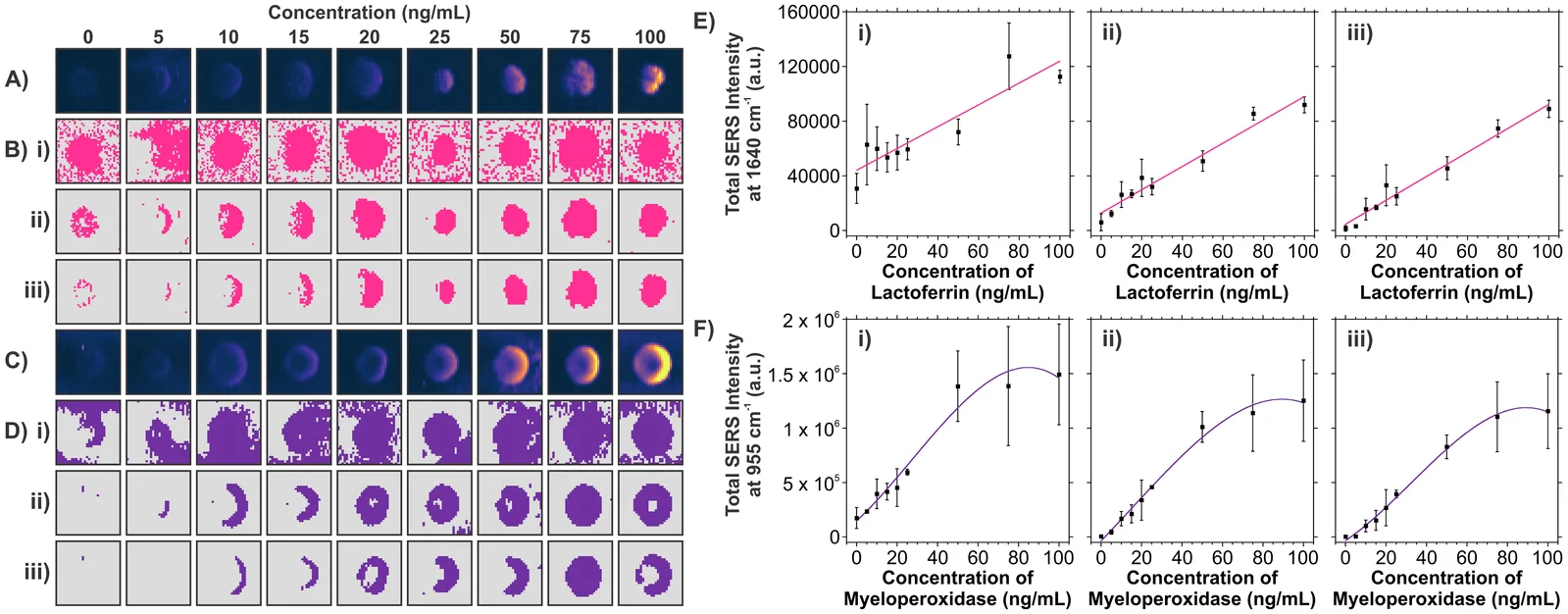
Construction of calibration curves using SERS images. (A) Heat maps of the SERS intensity at 1640 cm^−1^ for different concentrations of lactoferrin (LF). (B) Digital SERS-like reconstruction of the initial images using the signal intensity away from the spot as the background of the maps in A. Thresholds of (i) background, (ii) 2× background, and (iii) 3× background were used. (C) Heat maps of SERS intensity at 955 cm^−1^ for different concentrations of myeloperoxidase (MPO). (D) Digital SERS-like reconstruction of the initial images using the signal intensity away from the spot as the background of the maps in C. (E) Calibration curves for the quantification of lactoferrin concentration using the total SERS intensity of the active pixels as determined in B. A linear line of best fit was applied. (F) Calibration curves for the quantification of myeloperoxidase using the total SERS intensity of the active pixels in D. A third-order polynomial best fit was applied.

The outcomes from the decided paths of Fig. 2 are shown in Fig. 3. The SERS intensity heatmaps and binary SERS maps for one replicate of each concentration study are represented in Fig. 3A–D. To further establish the variable background threshold, the determined average background intensities were multiplied by values of 1 through 3. In effect, a value of background SERS intensity 3× should have similar efficacy as the signal being three times that as the noise. In doing so, pixels from outside the spot were no longer considered. Fig. 3E and F highlight how calibration curves can then change depending on what multiplication factor was used. From these calibration curves, and the results of Tables S3 and S4, the ideal combination was determined to be a threshold of 3× the background signal to establish the active and inactive pixels, coupled with the total SERS intensity from the spot to establish the concentration dependent intensity for the calibration curves. For LF, the linear range was found to be between 0 and 100 ng mL^−1^, with a limit of detection of 3.2 ng mL^−1^ and a limit of quantification of 18.8 ng mL^−1^ (corresponding to the average signal of the blank + 3× the standard deviation, and 10× the standard deviation, respectively). In comparison, the linear range for MPO was 5 to 75 ng mL^−1^. However, the limits of detection and quantification, based on the average signal and standard deviation of the 5 ng mL^−1^ samples, were 5.0 and 8.0 ng mL^−1^, respectively. The calibration curves also provide information about the effectiveness of the binding in the assay and saturation. Whilst the linear range of the LF calibration curve could be extended beyond what was evaluated in this work, the MPO calibration curve cannot. Above 75 ng mL^−1^, the curve saturates, and so a third order polynomial was applied to this dataset. Importantly, the dynamic linear ranges for both biomarkers are well below the normal concentrations found in stool; the accepted “normal” concentration for lactoferrin in stool is 7.25 µg g^−1^ (7250 ng mL^−1^) for both ulcerative colitis and Crohn's disease,^42^ and 60.34 µg g^−1^ (60 340 ng mL^−1^) and 22.2 µg g^−1^ (22 200 ng mL^−1^) for myeloperoxidase in the stool of ulcerative colitis and Crohn's patients, respectively.^43^ This is important because in a realistic assay, the stool sample would likely need to be processed, incorporate an extraction step, and then be diluted in a running buffer. This would result in the concentration of the biomarkers decreasing by at least tenfold, which would ideally be within the ranges examined in this concentration study, and allow for changes in concentration to be monitored before saturation of the calibration curve occurs. Further optimisation of the singleplex assays could then be carried out to establish effective calibration curves in the dynamic range of the completed assay.

### 3.3. Duplex detection of lactoferrin and myeloperoxidase

**Fig. 4:**
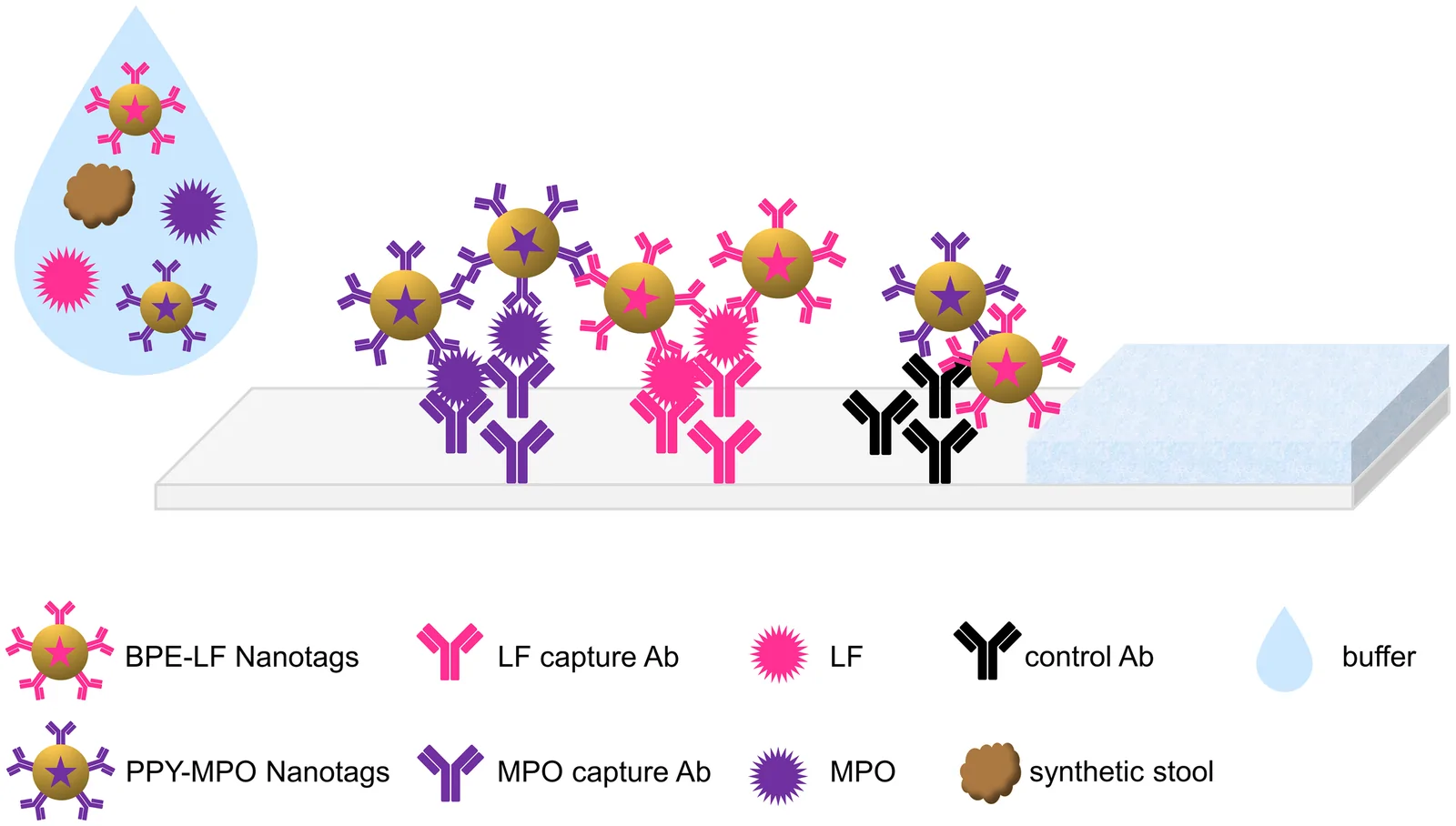
Schematic showing design of multiplexed lateral flow immunoassay; strips were prepared with MPO (purple) and LF (pink) test spots, and a goat anti-rabbit control spot. The strips were then dipped into a sample mixture containing buffer, synthetic stool, BPE-LF and PPY-MPO nanotags, and (i) no biomarkers, (ii) LF only, (iii) MPO only, or (iv) both LF and MPO.

The singleplex data confirmed that both biomarkers could be successfully detected using the dipstick assay. However, as highlighted in the introduction, monitoring IBD would be more informative if both biomarkers could be detected on the same strip, providing greater insight into disease progression. As already discussed, multiplexed SERS-LFIAs can be spatially or spectrally resolved. In short, there can be distinct test regions for each biomarker, with the possibility of using the same or different Raman reporters for each biomarker. Alternatively, a single combination test zone can be used, requiring unique Raman reporters for each biomarker. In this duplex assay, highlighted in Fig. 4, the best aspects of both approaches were used: two test spots were used to allow for straightforward visual analysis, and two distinct SERS nanotags were used to quantify the response, as well as allowing determination of contributions from non-specific interactions in each of the test spots. To evaluate whether both biomarkers could be simultaneously detected on the same strip, and to characterise the SERS signals from each test spot, four conditions were assessed: the absence of both biomarkers, MPO alone, LF alone, and an equal mixture of both biomarkers. The volumes of each component are shown in Table S2.

A comparison of the expected *vs.* visual outputs of the duplex LFIAs are presented in Fig. S12. In general, a spot was observed where expected, due to the biding between the biomarker and SERS tag. In some instances, even in the absence of the biomarker, there was a faint spot observed which was an indication of non-specific interactions. To establish potential origins of these interactions, and to ascertain if they were a hindrance to the SERS performance, the test spots were then Raman mapped using 785 nm laser excitation.

**Fig. 5:**
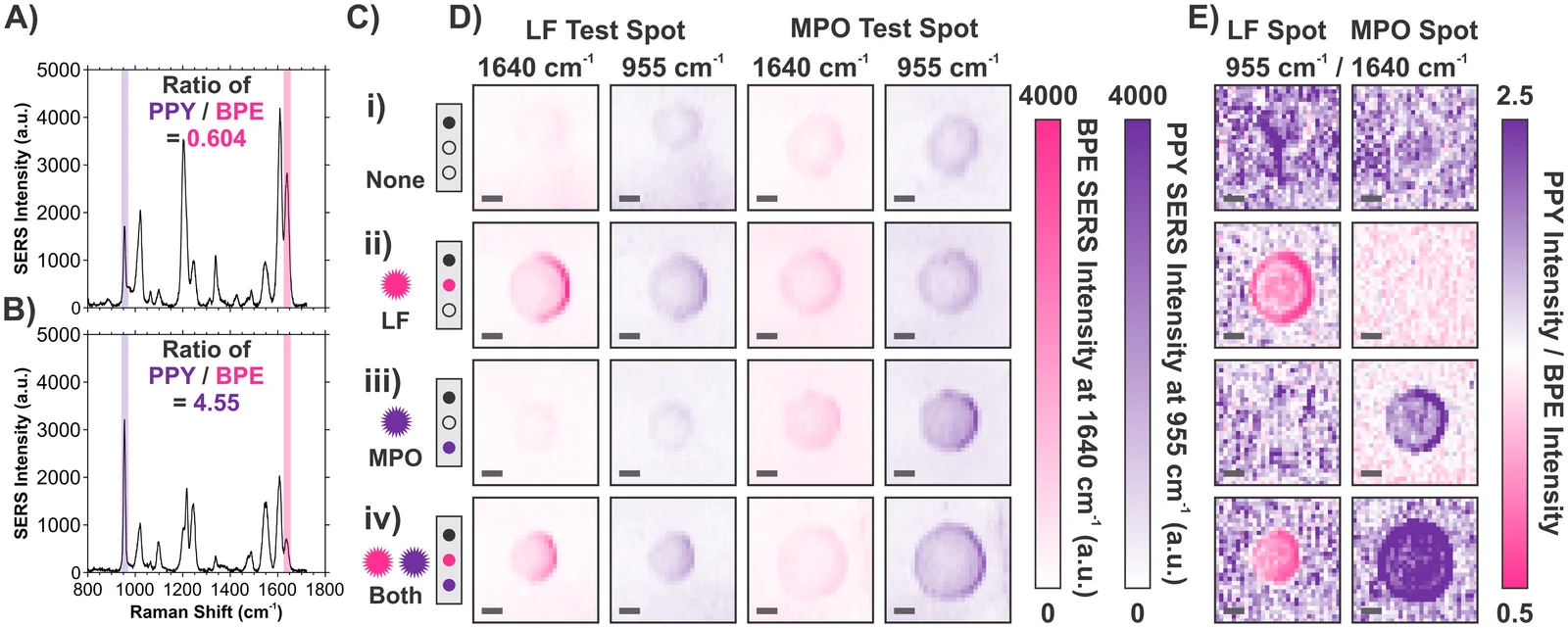
Analysis of the duplex SERS-LFIAs. (A) Representative SERS signal measured from the LF spot of the test run with LF present in the sample (BPE peak at 1640 cm^−1^ highlighted in pink, and PPY peak at 950 cm^−1^ highlighted in purple). (B) SERS signal measured from the MPO spot of the test run with MPO present in the sample. (C) Schematics representing the various samples containing (i) no biomarkers, (ii) LF, (iii) MPO, and (iv) LF and MPO. (D) Heatmaps of the SERS intensities for BPE and PPY in each of the test spots for the four duplex SERS-LFIAs. (E) False colour images created using the ratio of PPY/BPE (955 cm^−1^/1640 cm^−1^) to establish if a spot was truly positive for LF or MPO. Scale bars indicate 500 µm.

As with singleplex experiments, the SERS intensity of a characteristic peak from each Raman reporter was used to generate SERS heat maps. Here, it was important to evaluate the intensity of both reporters within each individual spot, to ascertain if both SERS nanotags were present due to non-specific interactions. This approach enabled us to assess whether the correct nanotag was binding to the appropriate test spot in the presence of its corresponding biomarker. The interpretations of the duplex SERS-LFIA are presented in Fig. 5.

It was anticipated that with the LF test spot, in an LF positive sample, only the SERS signal of the Raman reporter BPE would be observed. Likewise, in the MPO spot, for an MPO positive sample, only the SERS spectrum of PPE was expected. However, this was not observed (Fig. 5A and B). Instead, analysis revealed that both SERS nanotags were present at each spot regardless of the combination used (Fig. 5C and D), indicating non-specific binding and suggesting that the duplex lacked specificity. Closer inspection of the individual SERS spectra, however, showed a shift in the relative ratios of the two Raman reporters at each test spot. This is illustrated in Fig. 5A, where a SERS spectrum from the LF map demonstrated a larger contribution from the BPE signal, highlighted by the increased 1640 cm^−1^ peak, whilst the SERS signal from the MPO test spot had a larger PPY contribution, shown by the large peak at 955 cm^−1^ (Fig. 5B). We recognised that this change could be exploited to extract the correct information from each spot. Therefore, a second analysis was performed by calculating the ratio between characteristic Raman peaks. This ratiometric approach revealed a clear correlation between each Raman reporter and its corresponding biomarker test spot, demonstrating that simultaneous detection of both biomarkers was achievable.

To generate ratio maps, for each pixel within the map, the SERS intensity for one Raman reporter was divided by the SERS intensity of the other. Fig. 5E shows the resulting ratiometric maps corresponding to the SERS intensity at 955 cm^−1^/the SERS intensity at 1640 cm^−1^ (PPY/BPE), or alternatively, MPO/LF. Interestingly, in the absence of biomarkers, the ratio maps displayed no distinct test spots, even though the SERS intensity maps indicated that the nanotags were present. As such, this approach demonstrated that both nanotags were contributing sufficiently to the observed spot, and that by taking the ratio, it effectively eliminated the non-specific binding background signal observed previously. In the second scenario, where only LF was present in the sample, the corresponding ratio of MPO/LF within the LF test spot had a small value compared to the background. This indicated that in this area, the SERS intensity of the LF nanotags was sufficiently high enough to overcome the non-specific interactions of the MPO nanotags. As such, it is possible to state that LF was positively detected.

In contrast, no distinct features were detected on the MPO test spot in the sample absent of MPO. This indicates a greater contribution from the BPE nanotags at the LF spot, consistent with the correct nanotag binding and immobilising at its designated location. In the opposite scenario, when MPO was present, the only visible spot appeared at the MPO test spot, confirming that the PPY nanotag bound specifically in response to MPO. When both biomarkers were present, clear signals were observed in both test spots, with the LF test spot appearing as a small value in the ratio MPO/LF, and the MPO test spot appearing as a large value in the MPO/LF ratio.

Through this ratiometric approach, the influence of non-specific binding was eliminated, and the results demonstrated that each nanotag bound with enough specificity to its corresponding biomarker and test spot, as verified by the dominant ratio signal at each location. Unlike conventional spatially resolved duplex SERS-LFIA methods that employ only a single Raman reporter for multiple biomarkers, this strategy provides definitive evidence that the correct nanotag binds to the correct test spot without ambiguity, and eliminates the effects of non-specific binding, which has the potential to incorrectly increase the measured concentration of biomarker detected at each spot.

Given the success of the ratiometric approach, establishing a strategy for the duplex based on the approaches described in the singleplex measurement may provide success. By applying the same variable threshold rule of 3× the background for both Raman reporters, and defining an active pixel as one where the intensity for either reporter is greater than the threshold, the contributions from the background pixels can once again be omitted. A preliminary summary of this is shown in Fig. S13 and Table S4. This approach makes it clear that the greatest non-specific interactions are occurring with the MPO spots. This is likely due to the fact that these assays were carried out in a synthetic stool matrix rather than simply using running buffer, in order to more accurately simulate the interactions that would occur in a real world sample, and components of the synthetic stool matrix were likely contributing to non-specific binding at the test spots. However, under these conditions, we believe that suitable calibration curves could be generated, once the various concentrations and scenarios are considered. For the same concentrations studied in the singleplex experiments, this corresponds to 81 combinations, which in triplicate is 243 SERS-LFIAs. This further underpins the need for developing assay feasibility with low-cost approaches, such as spots, compared to lines.

## 4. Conclusions

This work demonstrates a series of proof-of-concept SERS based LFIAs that can quantify the levels of lactoferrin and myeloperoxidase in synthetic stool samples. These biomarkers were chosen as they are relevant to chronic conditions such as Crohn's disease and ulcerative colitis and are present in similar clinical ranges. Concentration studies were successfully carried out using Raman mapping for lactoferrin and myeloperoxidase in singleplex platforms. Although a low-cost approach to the assay development was used by using test spots rather than lines, the singleplex concentration studies showed considerable promise. By utilising the unique background SERS signal from each LFIA to establish a threshold, limits of detection were established of 3.8 and 8.0 ng mL^−1^. However, the linear dynamic range for LF was greater than for MPO.

Given the promising results of singleplex concentration studies, the two biomarkers were then detected in a spatially resolved duplex platform. For this method, mapping the SERS intensity alone provided little information, therefore ratiometric maps were created to gain more insight into the specificity of the assay. These showed that in most cases, the expected nanotags were bound to the test spots; although there were often higher than expected concentrations present due to the non-specific binding of the other nanotag. At this stage, multiplexing only provided a qualitative answer regarding the presence of each biomarker, and further work is required to make the multiplexed assay quantitative. Future work would include validating the assay using ELISAs and patient IBD samples, with the data obtained from the synthetic stool samples providing a useful foundation for further assay development.

Overall, the key innovation here is the establishment of a framework for the development and evaluation of single and duplex SERS-LFIA, enabling quantitative assessment of biomarkers through lower cost approaches. This provides a foundation for advancing robust, sensitive and quantitative point of care diagnostics platforms. As SERS-LFIA advances towards integration with portable, point and shoot spectrometers, with large laser spot sizes which can interrogate whole test spot or lines, it is essential to address challenges associated with background signals. The results here emphasise the importance of carefully interpreting spectra from multiple regions of the assay, rather than relying on single point measurements, and we must account for spatial variability and substrate interference to achieve optimal sensitivity. Point and shoot strategies should therefore incorporate strategies for robust background correction and multi-point spectral analysis to ensure reliable, high sensitivity detection at the point of care.

## Author contributions

Jodie Fergusson; investigation, data collection, assay development and optimisation, writing original draft. Sian Sloan-Dennison: assay development and optimisation, manuscript revisions. Gregory Q. Wallace: process and analysis of SERS maps, manuscript revisions. Sian Sloan-Dennison and Gregory Q. Wallace contributed equally. Neil C. Shand: funding acquisition. Karen Faulds and Duncan Graham: supervision, funding acquisition. All authors revised the final manuscript.

## Conflicts of interest

The authors declare no conflicts of interest.

## Supplementary Material

AN-151-D6AN00820H-s001

## Data Availability

All data is available at https://doi.org/10.15129/eb2467d3-0926-4566-9b3b-21bb3726a644. Supplementary information (SI) is available. See DOI: https://doi.org/10.1039/d6an00820h.
