# Supplementary material for: Analysis of multiplexed SERS-based lateral flow assays: turning qualitative into quantitative for inflammatory bowel disease monitoring
Source: Analyst. 2026 Aug 18;151(18):5439–48. doi: 10.1039/d6an00820h (PMC13491543; doi:10.1039/d6an00820h)
Supplement: Supplementary file 1 [file AN-151-D6AN00820H-s001.pdf]

Supplementary Information (SI) for Analyst.
This journal is © The Royal Society of Chemistry 2026

Analysis of Multiplexed SERS-based Lateral Flow Assays: Turning Qualitative into Quantitative for Inflammatory Bowel Disease Monitoring

Jodie Fergusson[1], Gregory Q. Wallace[1], Sian Sloan-Dennison[1], Neil C. Shand[2], Duncan Graham[1], Karen Faulds[1*]

1. Centre for Nanometrology, Department of Pure and Applied Chemistry, Technology and Innovation Centre, 99 George Street, Glasgow, G1 1RD
2. Defence Science and Technology Laboratory, Porton Down, Salisbury, SP4 0JQ

Supplementary Information

Table S1. Volumes of lateral flow buffer, synthetic stool matrix, biomarker, and nanotags used in singleplex concentration studies of lactoferrin and myeloperoxidase.

Final biomarker concentration (ng/mL)	Lateral flow buffer (μL)	Synthetic stool (μL)	Biomarker (μL)	Nanotags (μL)
**100.0**	77.5	10.0	10.0 (1 μg/mL)	2.5
**75.0**	80.0	10.0	7.5 (1 μg/mL)	2.5
**50.0**	82.5	10.0	5.0 (1 μg/mL)	2.5
**25.0**	85.0	10.0	2.5 (1 μg/mL)	2.5
**20.0**	67.5	10.0	20.0 (0.1 μg/mL)	2.5
**15.0**	72.5	10.0	15.0 (0.1 μg/mL)	2.5
**10.0**	77.5	10.0	10.0 (0.1 μg/mL)	2.5
**5.0**	82.5	10.0	5.0 (0.1 μg/mL)	2.5
**0.0**	87.5	10.0	0.0	2.5

Table S2. Volumes of lateral flow buffer, synthetic stool matrix, biomarker, and nanotags used in multiplexed detection of lactoferrin and myeloperoxidase.

Sample	Lateral flow buffer (µL)	Synthetic stool (µL)	LF (µL)	MPO (µL)	BPE-LF nanotags (µL)	PPY-MPO nanotags (µL)
**Blank**	85.0	10.0	0	0	2.5	2.5
**LF**	75.0	10.0	10	0	2.5	2.5
**MPO**	75.0	10.0	0	10	2.5	2.5
**LF + MPO**	65.0	10.0	10	10	2.5	2.5

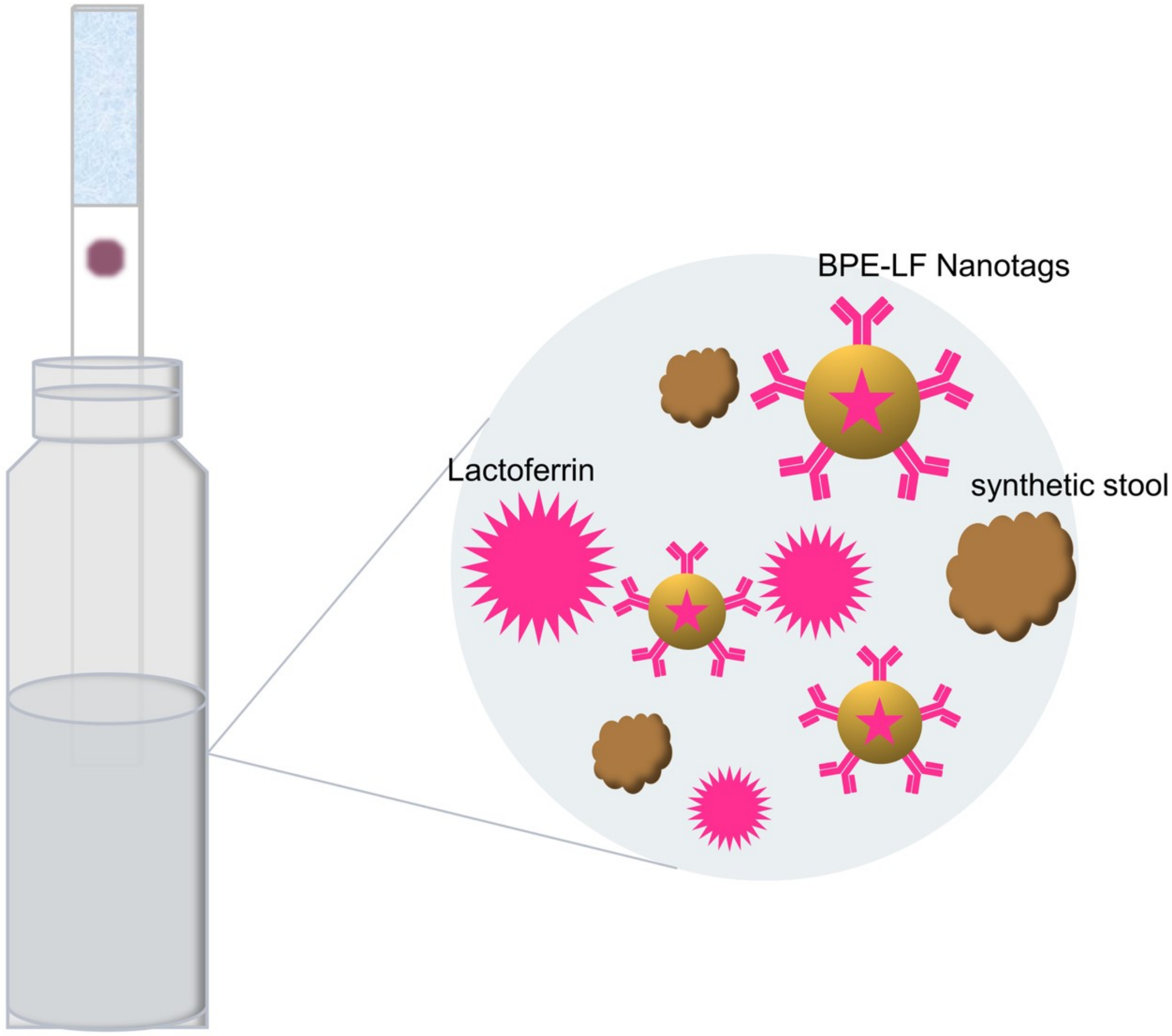

Figure S1. "Dipstick" sampling method used to analyse mixtures of nanotags, synthetic stool, and biomarkers.

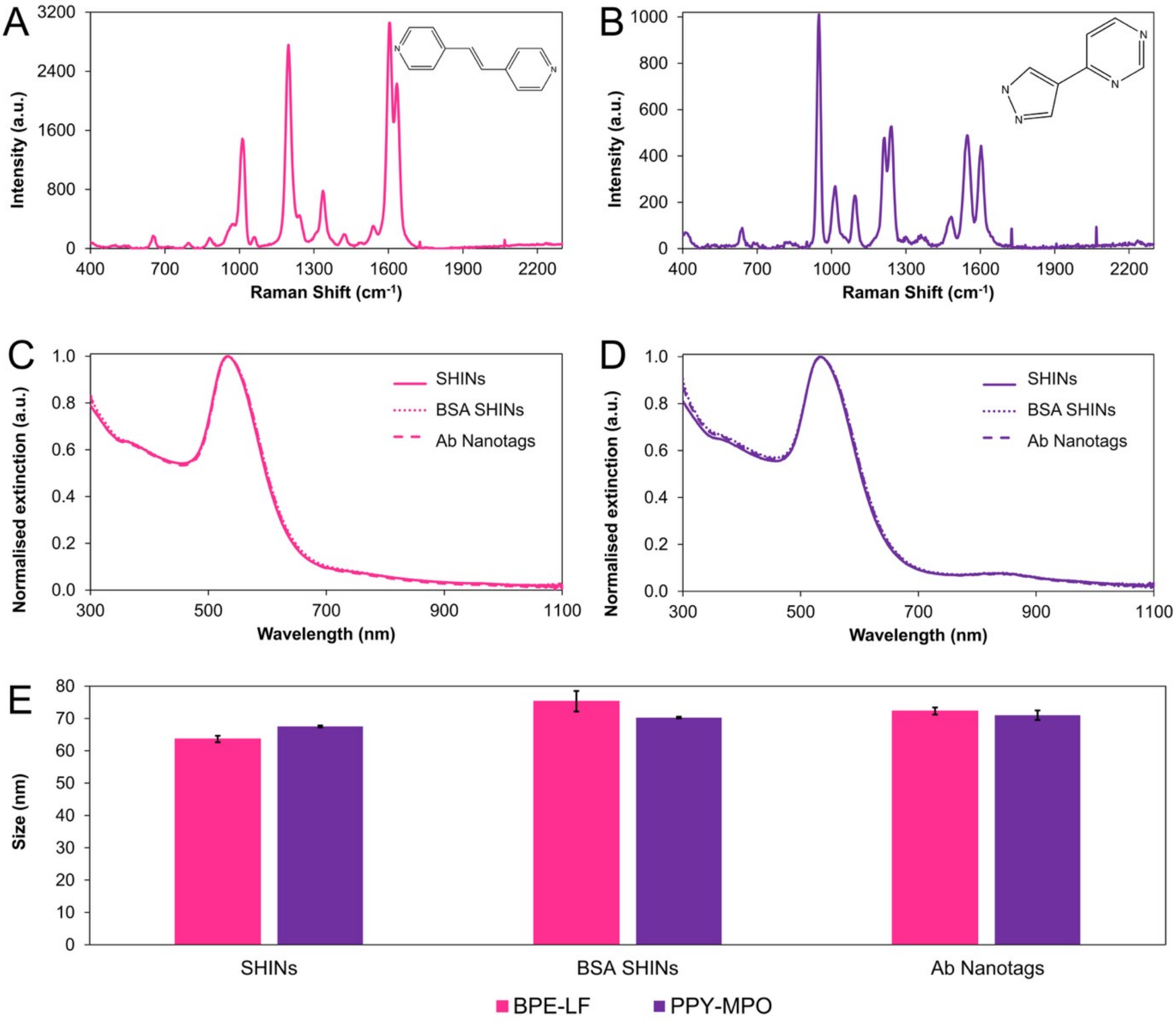

Figure S2. SERS spectra for (A) BPE-LF nanotags and (B) PPY-MPO nanotags; inset, the corresponding molecular structures. Extinction spectra for (C) BPE-LF and (D) PPY-MPO nanotags, functionalised with BSA and either lactoferrin or myeloperoxidase, normalised to the plasmon at ~ 530 nm for each sample. (E) Size measurements for BPE-LF and PPY-MPO nanotags functionalised with BSA, and either lactoferrin or myeloperoxidase. SERS measurements were collected using a Snowy Range Instruments handheld CBex spectrometer with 785 nm excitation wavelength, 10 mW laser power, and 0.1 s acquisition time. Each sample was scanned in triplicate, baseline corrected in MatLab (Version 2022b), and an average of the three scans plotted in Excel. Extinction measurements were collected using a Cary-60 UV-vis spectrophotometer, scanning from 300 nm to 1100 nm at a medium scan speed of 600 nm/min. Size measurements were obtained using a Malvern Nanoseries Zetasizer.

The characterisation data for the nanotags before and after antibody functionalisation are shown in Figure S2; even at the lowest possible laser settings of 0.1 s acquisition time and 10 mW laser power, the nanotags exhibit distinct spectra with minimal background noise. The BPE-LF nanotags have an intense peak at ~ 1610 cm^{-1}, and the PPY-MPO nanotags an intense peak at ~ 950 cm^{-1}, and these two peaks were used to monitor SERS intensity, and therefore biomarker concentration, in subsequent experiments. After functionalisation with the appropriate antibody, the nanotags were analysed using extinction spectroscopy and DLS. It was expected that if the surface had been successfully functionalised, there would be a redshift in the plasmon due to a change in the refractive index, and an increase in the hydrodynamic diameter, due to the overall increase in size. For BPE nanotags, the plasmon remained at 534 nm for both the LF-functionalised nanotags and BSA control; however, as the plasmon is largely dependent on the size of the metal core, the lack of a redshift does not necessarily indicate that the surface functionalisation was unsuccessful. The hydrodynamic diameter increased from 63.7 ± 1.0 nm to 75.3 ± 3.2 nm for the BSA control, and 72.3 ± 1.1 nm for the BPE-LF nanotags. The BSA control having a larger hydrodynamic diameter than the antibody-functionalisation was unexpected; it should be noted that the SOP used for DLS measurements was optimised for the refractive index of gold, and so the higher refractive indices of BSA and silica may have affected the accuracy of the readings. Alternatively, it may have simply been the case that during antibody functionalisation, the nanoparticle surface area remaining for functionalisation with BSA was reduced, and so less BSA was able to attach to the surface than in the case of the BSA control nanotags. Regardless, both the BSA control and LF-functionalised nanotags showed an increase in size from "bare" nanotags, indicating successful attachment of the antibody and BSA. The change in optical properties was more apparent for the PPY-MPO nanotags, with the plasmon redshifting from 532 nm for "bare" nanotags, to 535 nm for both the BSA control and the PPY-MPO nanotags, suggesting a small increase in size and therefore successful surface attachment. An increase in the hydrodynamic diameter was also observed, from 67.5 ± 0.3 nm for "bare" nanotags, to 70.3 ± 0.3 nm and 71.0 ± 1.5 nm for the BSA control and PPY-MPO nanotags, respectively. Again, this shows successful functionalisation of the antibody to the nanotag surface.

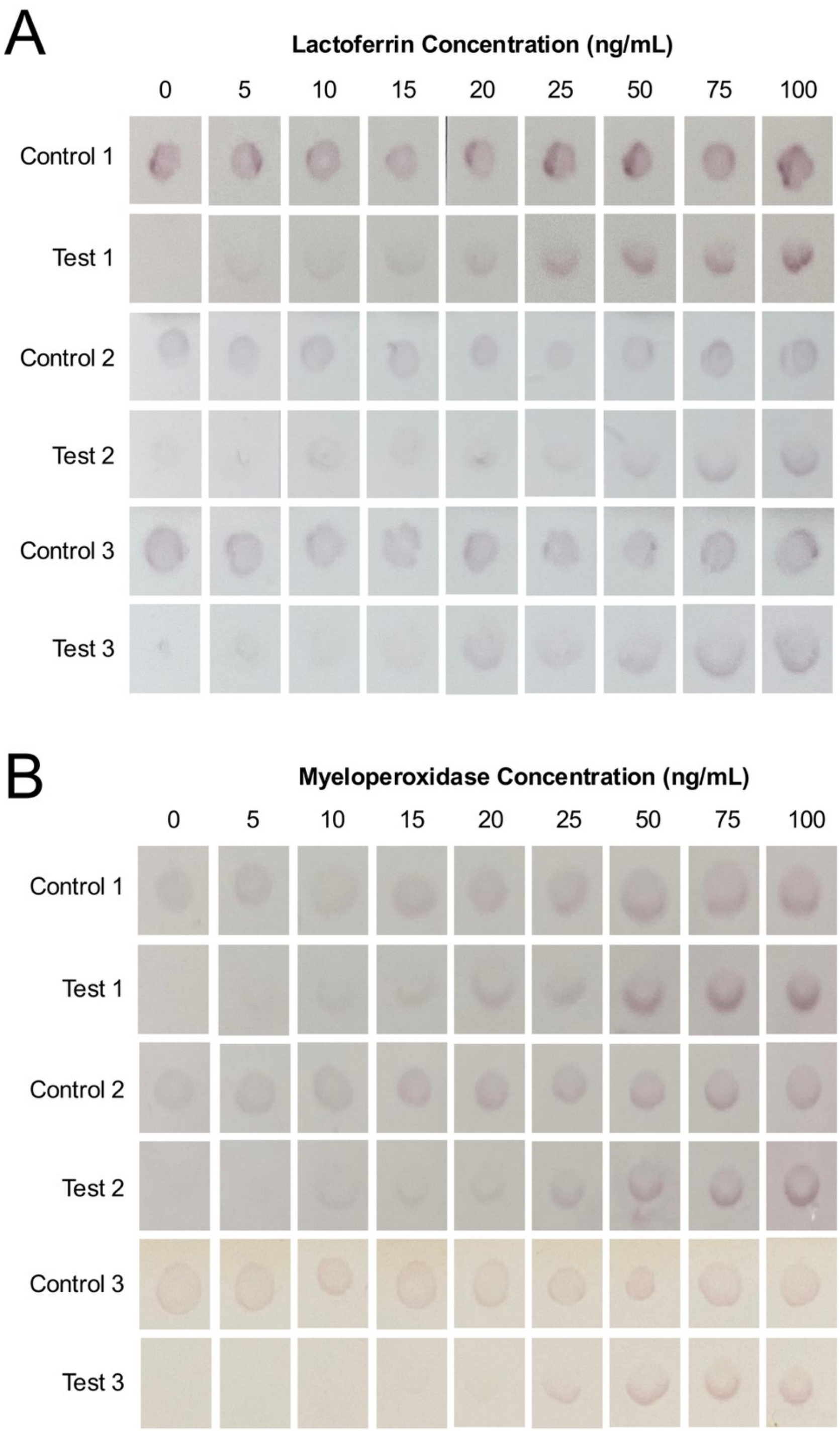

Figure S3. Optical images of test and control spots, obtained during triplicate concentration studies of (A) BPE-LF and (B) PPY-MPO LFIAs.

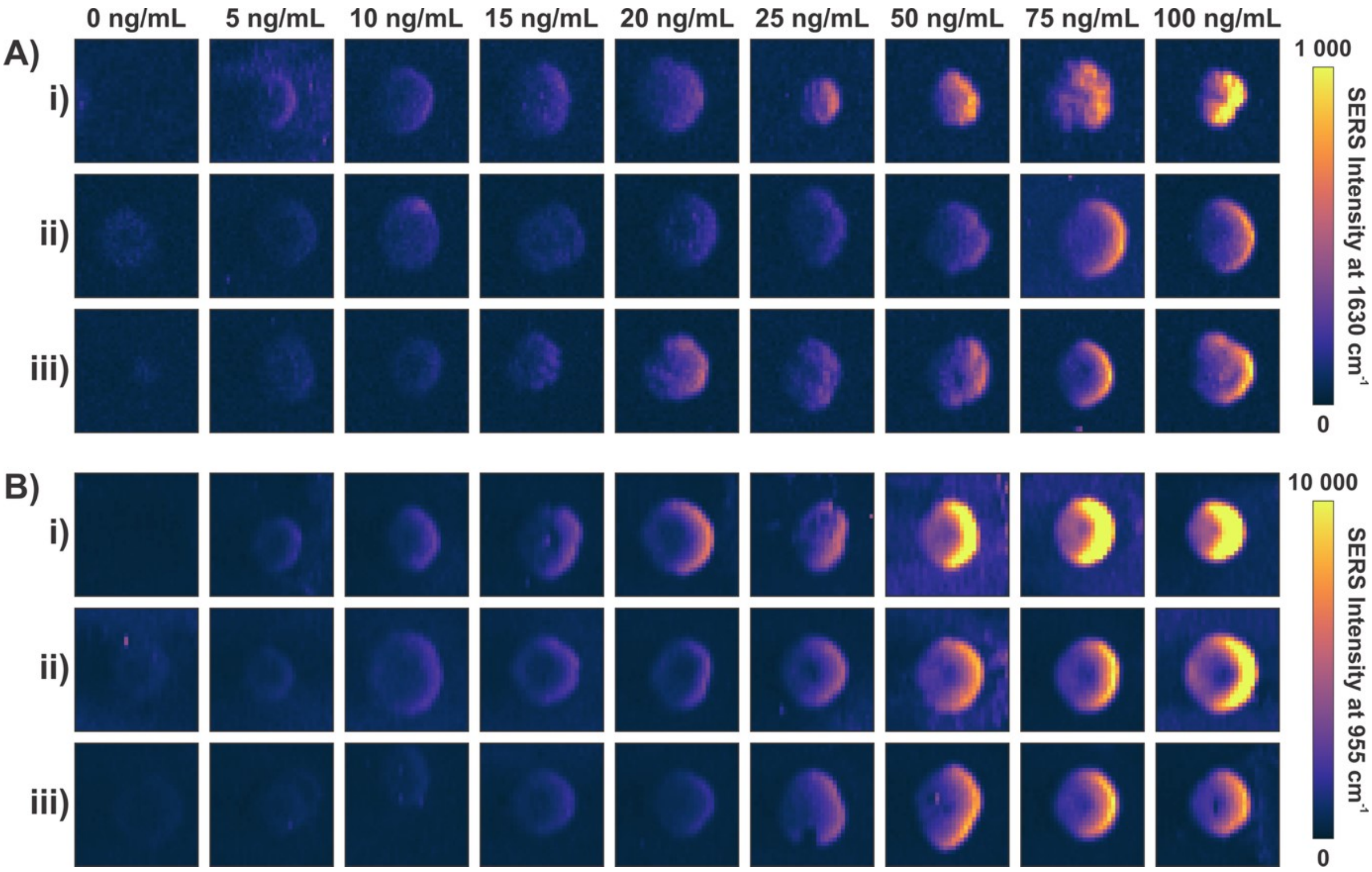

Figure S4. SERS intensity maps for triplicate concentration studies of (A) lactoferrin, and (B) myeloperoxidase.

Supplementary Note 1:

Within the dynamic SERS literature, spectra are often classified as being “active” if the intensity of the SERS signal is at least 3x greater than the noise, and/or if there are a defined number of peaks present within the spectrum. However, such a definition is less applicable in this work as the SERS nanotags can also non-specifically interact within any point of the map. A simpler method is to apply an intensity threshold across all pixels within the map. If the SERS intensity for the peak of interest is above that threshold, then the pixel would be classed as active. The challenge then becomes how best to define this threshold. In a previous study with a SERS LFIA,[1] the authors defined the threshold as being the average signal intensity, + 3x the standard deviation obtained from the test line of the negative control. This can then be thought of as applying a fixed value threshold to all spectra. Figure S5 shows the conversion of the SERS maps to binary maps, where pixels are classified as being active or inactive. A comparison between two thresholds (600 a.u., 1000 a.u.) clearly shows how the distribution of active and inactive pixels can change. This can also be observed in the example for Method 1 in Figure 2. Importantly, these binary maps also indicate that pixels located outside of the test spot will be classified as being active and will therefore contribute when the calibration curves are generated. To better account for this, five regions were selected from each map: the four corners, and an area in front of the test spot (example for Method 2 in Figure 2, and Figure S6A). However, as shown in Figure S6B, the background SERS signals vary for each SERS LFIA. As such, relying on a fixed value, even if it were derived from a negative control, does not adequately account for the different amounts of non-specific interactions that occur on each LFIA.

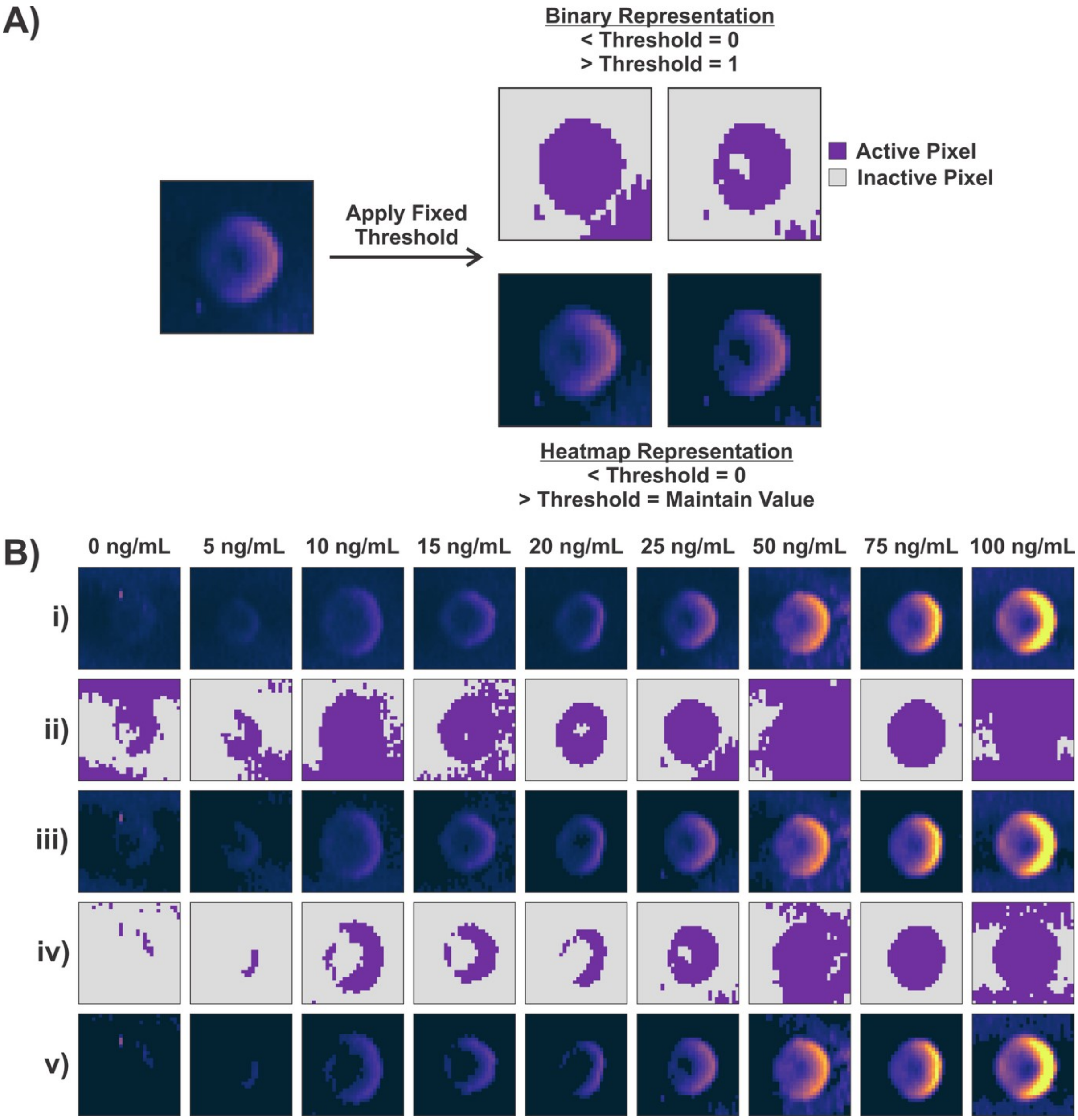

Figure S5. (A) General strategies for visualising SERS maps after thresholding using a binary representation that shows the active and inactive pixels, as well as a heatmap representation that keeps the SERS intensities for pixels where the SERS intensities are above the threshold. Two thresholds (600 a.u. and 1000 a.u.) are shown. (B) Representative outputs of thresholding on the SERS maps for one concentration series of myeloperoxidase; i) a threshold of 600 a.u., ii) binary map, iii) heatmap with a threshold of 1000 a.u., iv) binary map, and v) heatmap.

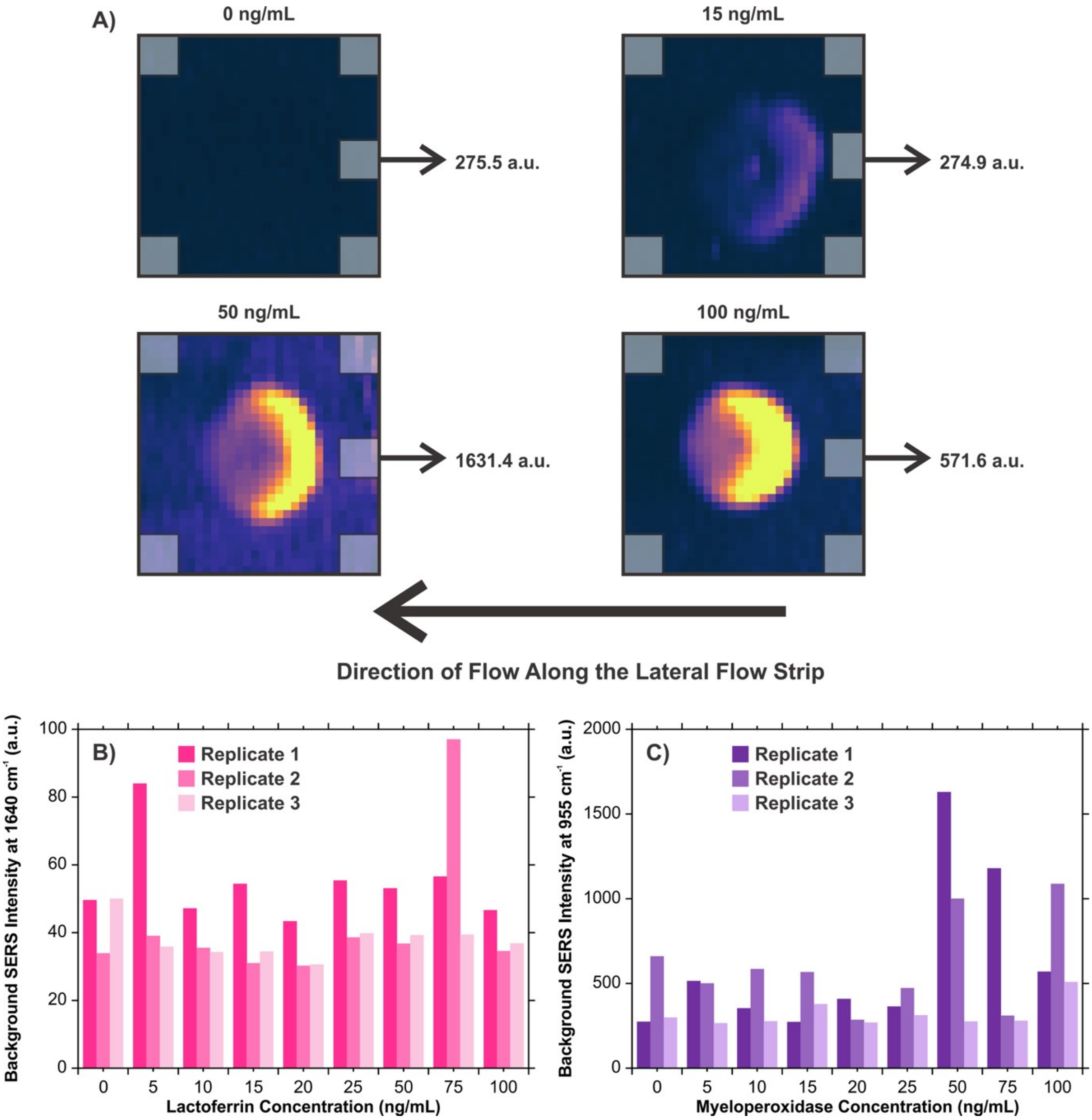

Figure S6. (A) Representative schemes for quantifying the background signal. From each map, 5 areas of 5 x 5 pixels were selected: the four corners, and an area in front of the test spot. The values reported corresponded to the average intensity of those areas. The background SERS signals for the replicates of the concentration studies of (B) lactoferrin and (C) myeloperoxidase.

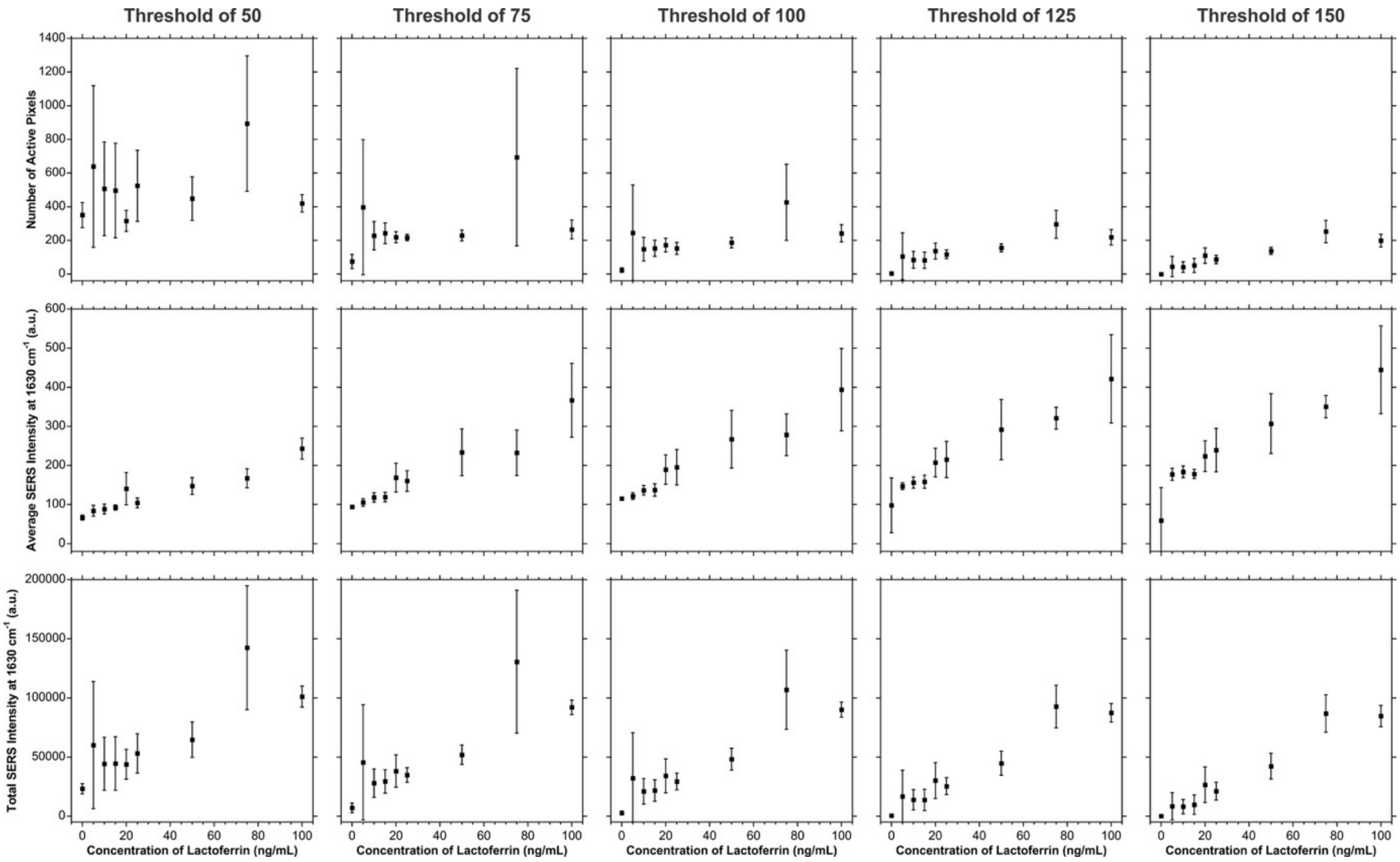

Figure S7. Calibration curves for lactoferrin using various fixed thresholds and quantification strategies.

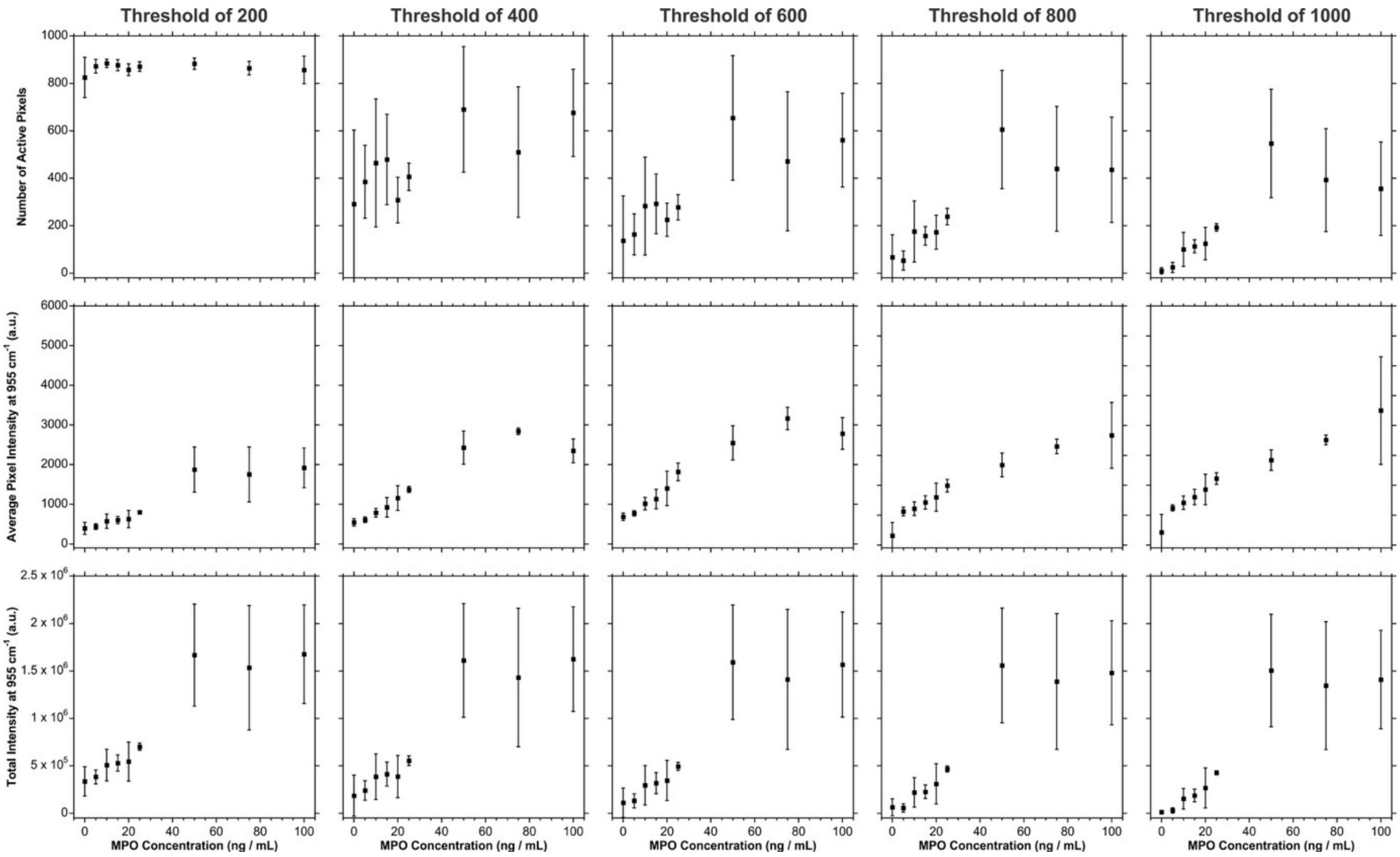

Figure S8. Calibration curves for myeloperoxidase using various fixed thresholds and quantification strategies.

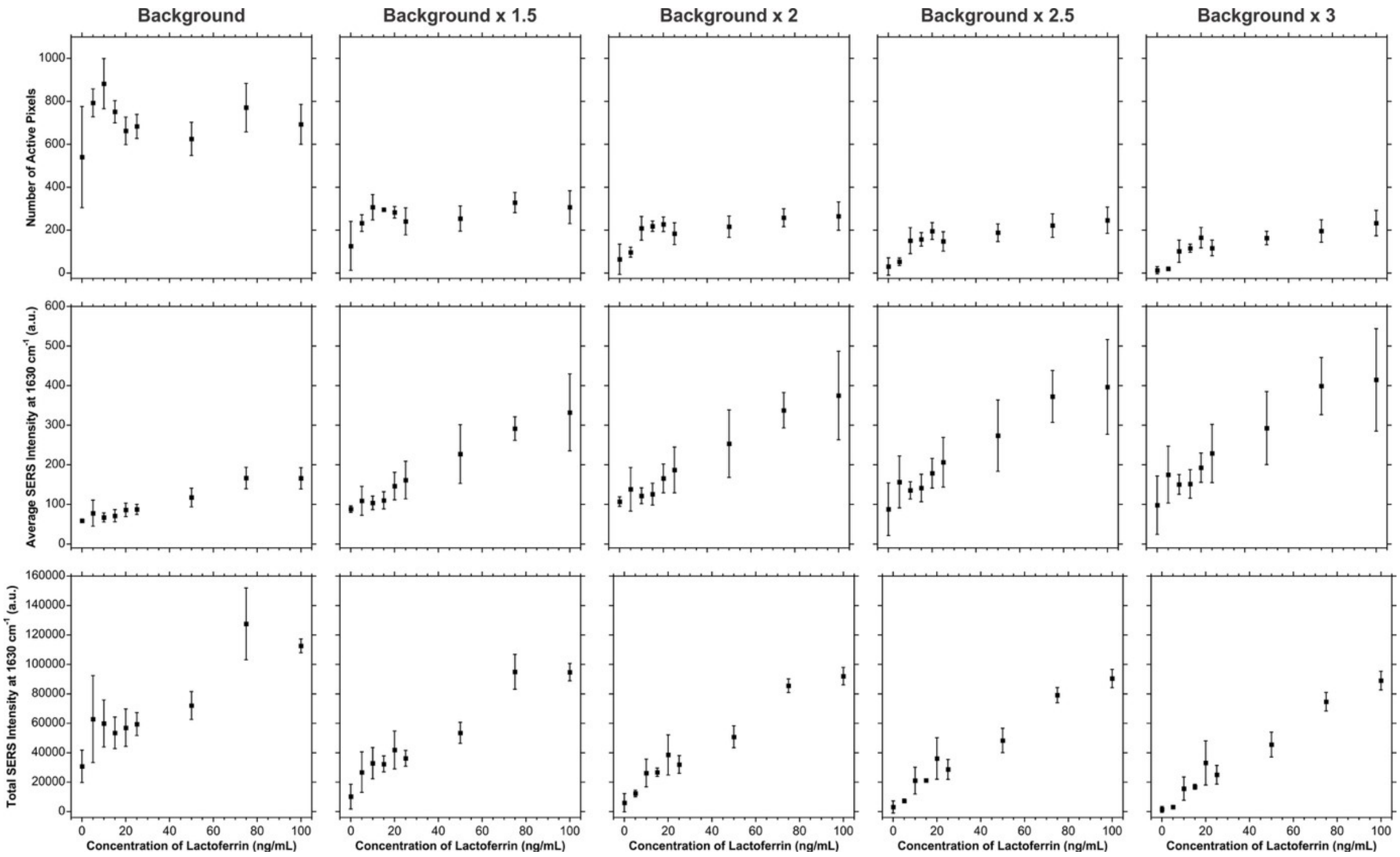

Figure S9. Calibration curves for lactoferrin using the background SERS intensity, multiplied by various values to create the variable thresholds.

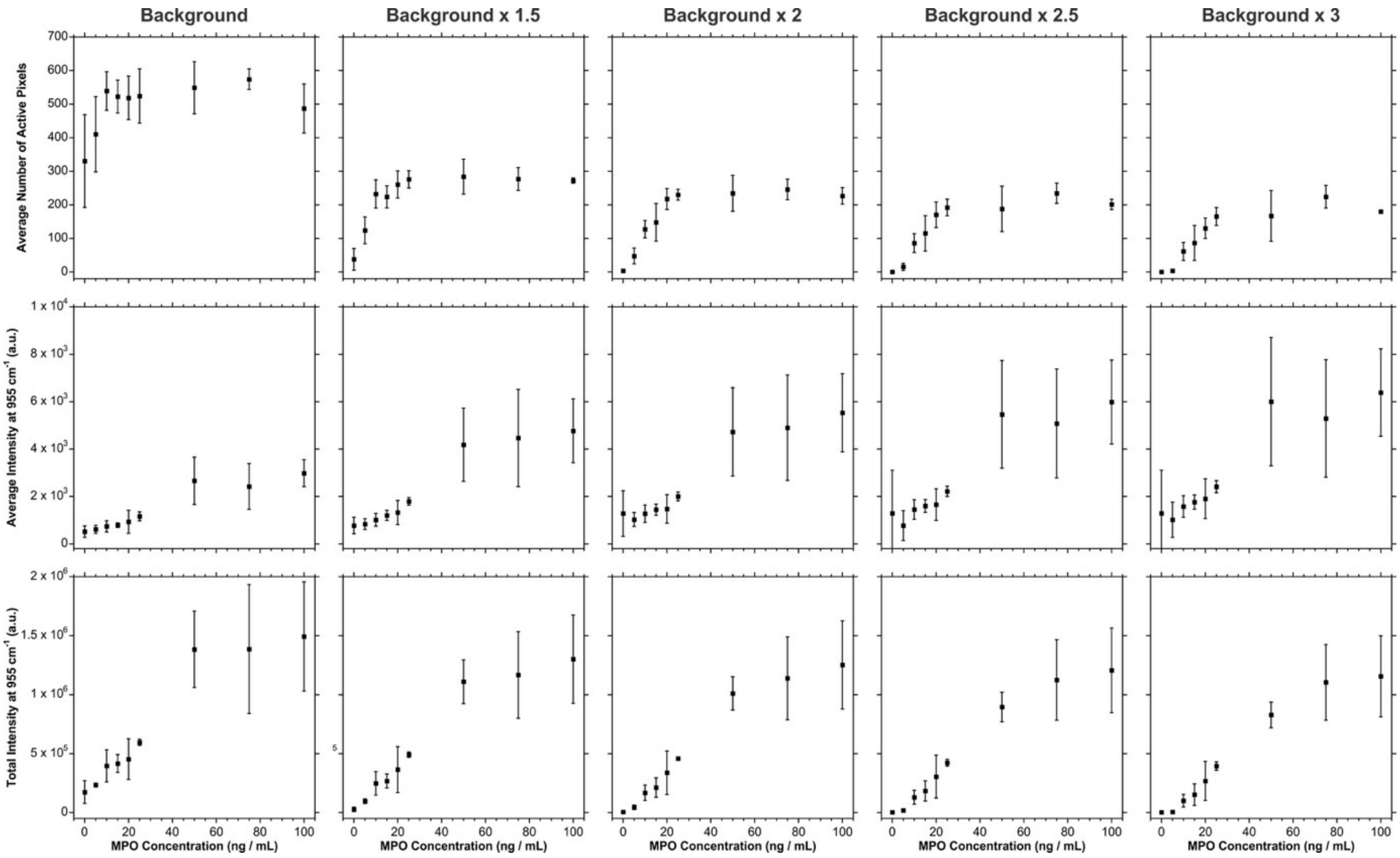

Figure S10. Calibration curves for myeloperoxidase, using the background SERS intensity multiplied by various values to create the variable thresholds.

Table S3. Analysis of the calibration curve methods and strategies for quantifying lactoferrin. The number of pixels was not evaluated due to the poor calibration results.

Average Pixel Intensity					
**Threshold**	**Linear Range (ng / mL)**	**R^2**	**RSD at 50 ng/mL (%)**	**LOD (ng / mL)**	**LOQ (ng / mL)**
50 a.u.	0 – 100	0.911	17.8	10.1	46.7
75 a.u.	0 – 100	0.936	31.4	0.553	3.04
100 a.u.	0 – 100	0.959	33.9	4.0	13.7
125 a.u.	0 – 100	0.967	32.2	78.4	284.3
150 a.u.	5 – 100	0.982	30.5	75.0	322.6
Background	0 – 75	0.956	24.4	16.6	42.8
Background × 1.5	0 – 75	0.984	39.9	11.9	37.4
Background × 2	0 – 75	0.973	41.0	15.9	49.7
Background × 2.5	0 – 75	0.966	40.4	64.4	227.0
Background × 3	0 – 75	0.956	38.6	67.9	239.6
**Average Total Intensity**					
**Threshold**	**Linear Range (ng/mL)**	**R^2**	**RSD at 50 ng/mL (%)**	**LOD (ng/mL)**	**LOQ (ng/mL)**
50 a.u.	0 – 100	0.707	28.3	4.8	46.2
75 a.u	0 – 100	0.736	19.3	2.7	39.8
100 a.u.	0 – 100	0.854	23.3	6.7 *	20.4 **
125 a.u.	0 – 100	0.928	28.0	2.2 *	6.5 **
150 a.u.	0 – 100	0.946	31.6	0.73 *	2.2 **
Background	0 – 100	0.821	16.0	34.0	151.9
Background × 1.5	0 – 100	0.933	16.4	26.0	113.0
Background × 2	0 – 100	0.957	18.0	19.0	81.4
Background × 2.5	0 – 100	0.967	20.9	11.5	52.6
Background × 3	0 – 100	0.972	22.7	4.7	23.8

LOD and LOQ calculations utilised the average intensity of the blank, + 3x and 10x the standard deviation of the blank, respectively, to determine a value, that was then compared to the line of best fit to determine the concentration.

* LOD was calculated by omitting the y-intercept and only considering the slope.

** LOQ was calculated by omitting the y-intercept and only considering the slope.

Table S4. Analysis of the calibration curve methods and strategies for quantifying myeloperoxidase. The number of pixels was not evaluated due to the poor calibration results.

Average Pixel Intensity					
**Threshold**	**Linear Range (ng/mL)**	R^2	**RSD at 50 ng/mL (%)**	**LOD (ng/mL)**	**LOQ (ng/mL)**
200 a.u.	0 – 100	0.872	37.3	30.8	105.1
400 a.u.	0 – 75	0.979	21.1	11.4	34.7
600 a.u.	0 – 75	0.979	20.7	9.3	32.4
800 a.u.	5 – 100	0.973	18.2	20.7 *	64.9 *
1000 a.u.	5 – 100	0.990	14.8	13.5 *	39.1 *
Background	0 – 100	0.903	46.0	32.6	109.7
Background × 1.5	0 – 100	0.916	45.2	28.9	92.5
Background × 2	0 – 100	0.915	48.4	75.8	236.4
Background × 2.5	0 – 100	0.887	50.9	124.9	403.0
Background × 3	0 – 100	0.876	55.3	116.6	381.4
**Average Total Intensity**					
**Threshold**	**Linear Range (ng/mL)**	R^2	**RSD at 50 ng/mL (%)**	**LOD (ng/mL)**	**LOQ (ng/mL)**
200 a.u.	0 – 100	0.862	39.5	35.1	120.8
400 a.u.	0 – 100	0.859	45.5	46.8	160.0
600 a.u.	0 – 100	0.852	46.5	32.3	110.5
800 a.u.	5 – 75	0.860	47.5	11.5	28.7
1000 a.u.	5 – 75	0.864	48.3	9.7	20.6
Background	0 – 100	0.898	28.7	19.8	75.6
Background × 1.5	0 – 75	0.945	20.6	2.4	11.6
Background × 2	0 – 75	0.963	17.0	0.8	2.8
Background × 2.5	5 – 75	0.980	17.1	6.2 *	14.1 **
Background × 3	5 – 75	0.988	16.1	5.3 *	9.0 **

LOD and LOQ calculations utilised the average intensity of the blank, + 3x and 10x the standard deviation of the blank, respectively, to determine a value, that was then compared to the line of best fit to determine the concentration.

* LOD was calculated using the 5 ng/mL results instead of the 0 ng/mL results.

** LOQ was calculated using the 5 ng/mL results instead of the 0 ng/mL results.

Supplementary Note 2:

From the calibration curve results of Figures S7-S10, and Tables S3 and S4, it was apparent that using the number of active pixels was an ineffective approach. This was unsurprising, as the sizes of test spots varied and were independent of the biomarker concentration. As such, this metric was deemed incompatible for quantification. Average SERS intensity compared to total SERS intensity was often comparable, with different metrics having different effectiveness depending on the method of determining active and inactive pixels. Furthermore, there were instances where the constant thresholds may appear to have outperformed the variable threshold when some parameters were considered. Linear regression analysis provided greater insights (Figure S11). These showed that the confidence intervals, as well as the distribution of data points, were better for the variable threshold compared to the fixed threshold. Ultimately, it was decided that as the variable threshold approach resulted in fewer pixels from the outside of the spot contributing to the average or total SERS intensity, this would be the ideal method going forward. Using this method, the best combination of maximising the R2, minimising the relative standard deviation, minimising the LOD, and minimising the LOQ, were obtained using the total SERS intensity of the active pixels.

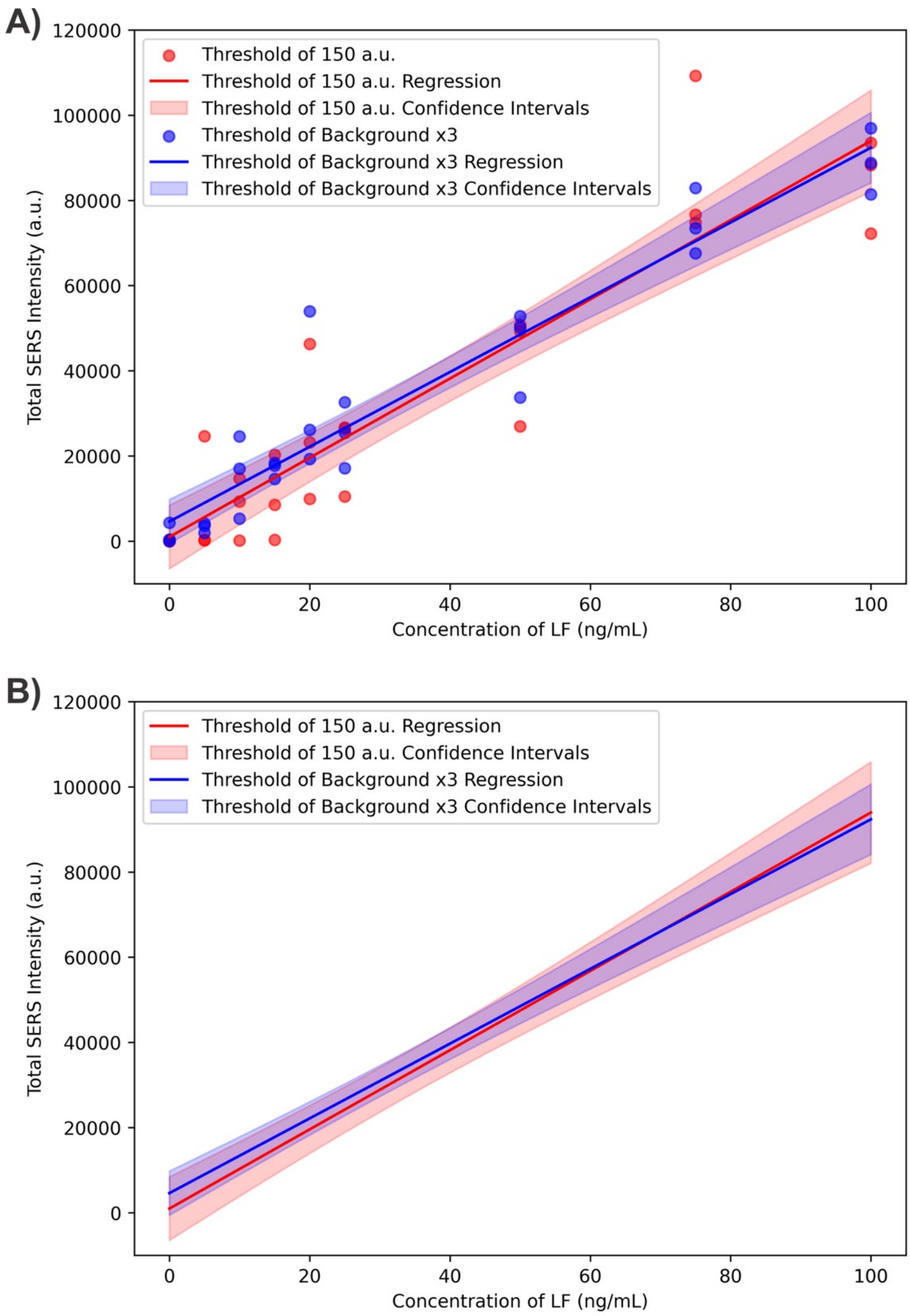

Figure S11. Linear regression for calibration curves of lactoferrin considering a threshold of 150 a.u., compared to a threshold of 3x the background. For clarity, (A) contains the data points, and (B) has them omitted. Confidence intervals of 95% are shown.

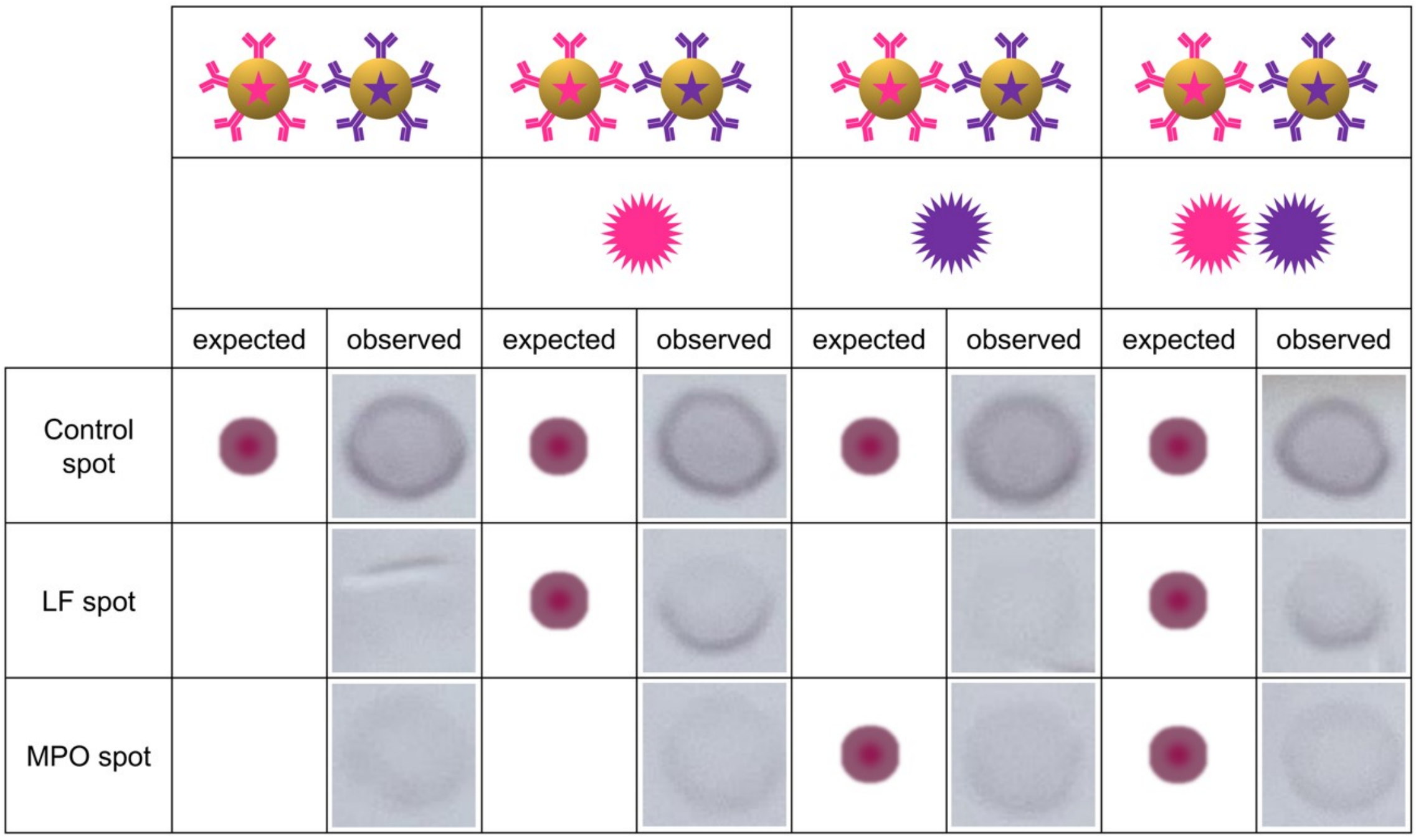

Figure S12. Comparison of expected vs observed presence of spots after running various duplex SERS LFIAs. BPE-LF nanotags and LF in the sample mixture are represented in pink; PPY-MPO nanotags and MPO in the sample mixture are represented in purple.

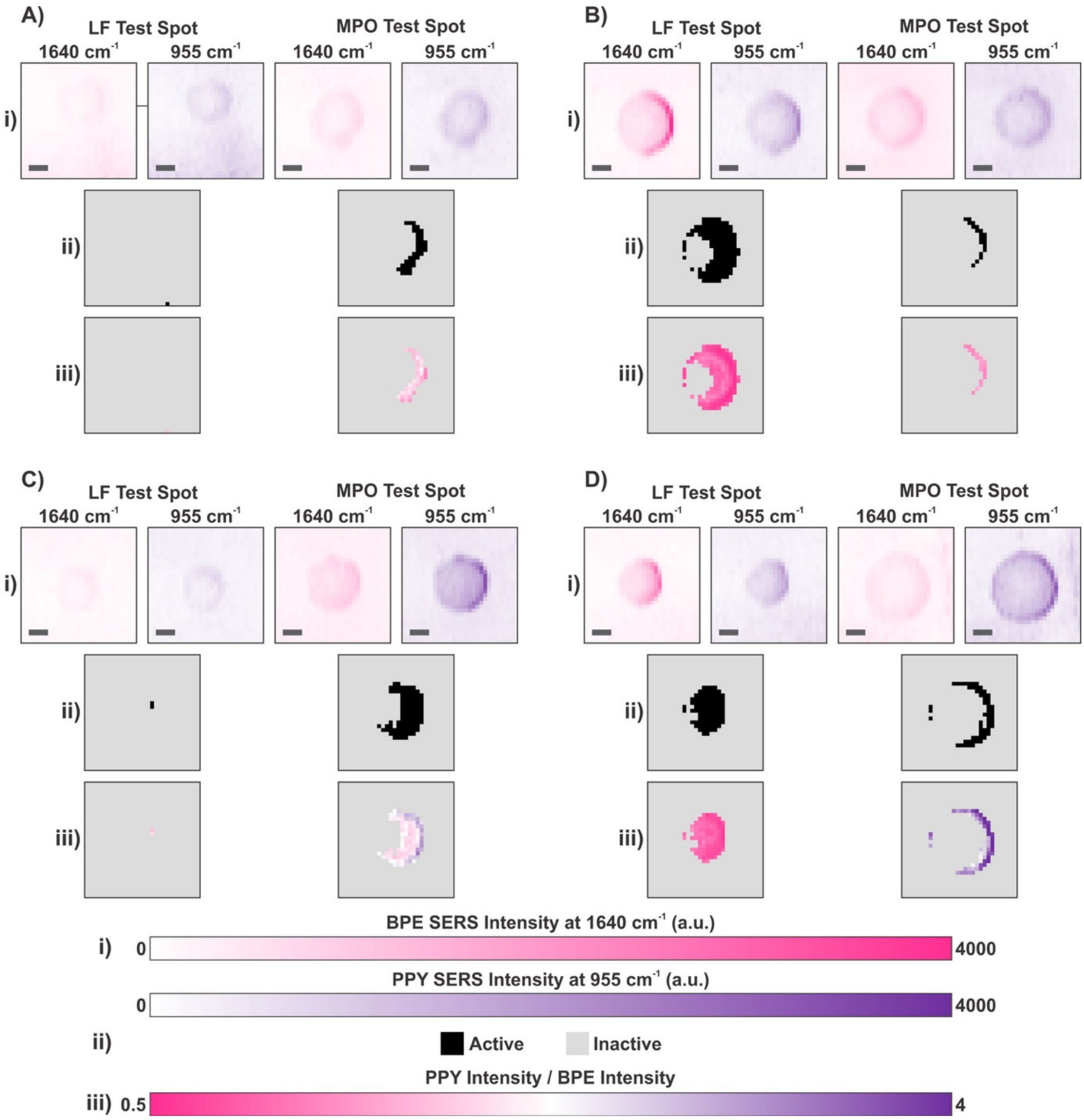

Figure S13. Threshold analysis and ratio value analysis of active pixels in the duplex SERS-LFIAs containing (A) no biomarkers, (B) LF only, (C) MPO only, and (D) both biomarkers. I) SERS intensity maps, ii) map of active pixels, and iii) map of PPY-MPO intensity/BPE-LF intensity for the active pixels.

References

1. W. Wang, S. Srivastava, A. Garg, C. Xiao, S. Hawks, J. Pan, N. Duggal, G. Isaacman-VanWertz, W. Zhou, L. C. Marr and P. J. Vikesland, *Environ. Sci. Technol.*, 2024, **58**, 4926-4936.
